# Granular cytoplasmic inclusions in astrocytes and microglial activation in the fetal brain of pigtail macaques in response to maternal viral infection

**DOI:** 10.1186/s40478-025-01970-9

**Published:** 2025-03-11

**Authors:** Raj P. Kapur, Andrew E. Vo, Amanda Li, Miranda Li, Jeff Munson, Hazel Huang, Briana Del Rosario, Orlando Cervantes, Hong Zhao, Ashley Vong, Gygeria Manuel, Edmunda Li, Monica Devaraju, Xuemei Deng, Audrey Baldessari, W. McIntyre Durning, Solomon Wangari, Brenna Menz, Audrey Germond, Chris English, Michelle Coleman, Austyn Orvis, Sidney Sun, Ed Parker, Sandra Juul, Brendy Fountaine, Lakshmi Rajagopal, Kristina M. Adams Waldorf

**Affiliations:** 1 Department of Laboratory Medicine and Pathology, Seattle Children’s Hospital, Seattle, WA USA; 2 Department of Laboratory Medicine and Pathology, University of Washington, Seattle, WA USA; 3 Department of Obstetrics and Gynecology, University of Washington, Seattle, WA USA; 4 Case Western Reserve University, Cleveland, OH USA; 5 School of Medicine, University of Washington, Seattle, WA USA; 6 Department of Psychiatry, University of Washington, Seattle, WA USA; 7 Department of Global Health, University of Washington, Seattle, WA USA; 8 Washington National Primate Research Center, Seattle, WA USA; 9 Center for Global Infectious Disease Research, Seattle Children’s Research Institute, Seattle, WA USA; 10 Department of Ophthalmology, University of Washington, Seattle, WA USA; 11 Department of Pediatrics, University of Washington, Seattle, WA USA

**Keywords:** Pregnancy, Fetus, Neurodevelopment, Astrocyte, Microglia, Lysosomal activation, Virus, Influenza a virus, Zika virus, Glial, Autophagy, Prenatal, White matter

## Abstract

The fetal origins of neuropsychiatric disorders are poorly understood but have been linked to viral or inflammatory injury of the developing brain. The fetal white matter is particularly susceptible to injury as myelination, axonal growth, and deep white matter tracts become established. We have used the pigtail macaque (*Macaca nemestrina*) to study the maternal and fetal effects of influenza A virus (FLUAV) and Zika virus (ZIKV) infection during pregnancy, in cohorts with different time intervals between inoculation and delivery. We observed a striking histopathological alteration in a subset of astrocytes which contained granular cytoplasmic inclusions (“inclusion cells”, ICs) within a specific region of the deep cerebral white matter in the fetal brains from specific FLUAV and ZIKV cohorts. Immunohistochemical and ultrastructural characteristics of ICs indicated that they are astrocytes (GFAP+) undergoing autophagocytosis (p62+) with activated lysosomes (LAMP1+, LAMP2+) and reactive changes in neighboring microglia. There was also a positive correlation between the number of ICs and LAMP1 or LAMP2 immunoreactivity in the fetal brain (LAMP1: rho 0.66; LAMP2: rho 0.54, *p* < 0.001 for both). Interestingly, ICs were significantly more prevalent in the 5-day FLUAV cohort and the 21-day intermediate ZIKV cohort than in controls (*p* < 0.005 and *p* = 0.04, respectively), but this relationship was not apparent in the ZIKV cohort with a shorter (2–3 days) or longer (months) time course. Virologic and immunologic assays indicated that the appearance of these cells was not linked with fetal brain infection. ICs were not observed in a macaque model of perinatal hypoxic ischemic encephalopathy. These alterations in fetal white matter are pathologically abnormal and may represent a transient neuropathologic finding that signifies a subtle brain injury in the fetus after maternal viral infection.

**Supplementary Information:**

The online version contains supplementary material available at 10.1186/s40478-025-01970-9.

## Introduction

Neurodevelopment spans fetal life, childhood, and adolescence which represents an extended period over which the developing brain is vulnerable to injury. Viral injury to the brain during fetal life can have particularly devastating consequences and result in a wide range of structural, neurocognitive, and motor deficits. Given the complexity of the human central nervous system and the long prenatal period over which neurodevelopment proceeds, the fetal brain is vulnerable to subclinical sources of injury which may leave subtle anatomical traces that may yield potentially significant effects on neurological outcome. Many patients with cognitive or neurobehavioral and neuropsychiatric disorders (e.g., autism, developmental delay, schizophrenia, epilepsy) do not exhibit major gross or dramatic microscopic alterations in brain anatomy; although some cases demonstrate subtle histological, molecular or circuitry changes, which may play a role in the pathogenesis of these conditions [1–3]. For many of these disorders, prenatal injury to the developing brain has been hypothesized as an antecedent event, although the nature of the injurious process is unknown.

White matter in the brains of preterm infants is particularly susceptible to a variety of injurious etiologies including maternal infections, which may or may not cross the placenta to infect the fetus and cross the fetal blood-brain barrier [4]. Even non-infectious processes that may be influenced by differences in maternal immune status appear to impact on fetal brain development and affect neurodevelopment and the risk for psychiatric illness long after birth [5]. These adverse effects on white matter development are particularly prevalent during the late second and third trimesters when axonal growth, early myelination, and deep white matter tracts are established [6, 7]. Many factors are believed to contribute to the vulnerability of fetal white matter including the incomplete state of development of the vascular supply, localized damage caused by free radicals, and toxic cytokines induced in response to maternal and/or fetal infection or inflammation [6]. Key cellular mediators of white matter injury include reactive astrocytes and “activated” microglia [8, 9].

As part of two independent research projects, we have used the pigtail macaque (*Macaca nemestrina*) as a model to study the maternal and fetal effects of influenza A virus (FLUAV) and Zika virus (ZIKV) infection during pregnancy. Included in these models are pregnancies in which the dam was inoculated subcutaneously with ZIKV late in the first trimester and fetuses, delivered by Cesarean section, and were necropsied in the mid-to-late third trimester. Prenatal magnetic resonance imaging and histology of some of the latter fetal brains provided evidence for damage to the ventricular lining and periventricular gliosis [10, 11], in addition to histologically subtle, but ultrastructurally and molecularly profound, defects in oligodendrocyte maturation and myelination [12]. In expanding these studies to include shorter intervals between inoculation and delivery, as well as FLUAV-infected dams, we observed an unusual and dramatic histopathological alteration of astrocytes primarily in a specific region of the deep cerebral white matter. Herein, we describe the light and electron microscopic characteristics of these cells and provide evidence that they are associated with astrocyte autophagocytosis and lysosomal activation, as well as reactive changes in neighboring microglia. These changes are independent of fetal brain infection, pursue a different time course after maternal inoculation with FLUAV versus ZIKV, and may affect brain development.

## Methods

### Ethical approvals

All animal experiments were carried out in strict accordance with the recommendations in the Guide for the Care and Use of Laboratory Animals of the National Research Council and the Weatherall report, “The use of non-human primates in research”. Animals were housed and experiments performed at the Washington National Primate Research Center (WaNPRC). The University of Washington Institutional Animal Care Use Committee (IACUC) approved the protocol (Permit Numbers: 4165-02, 4165-03, 3328-05). All surgery was performed under general anesthesia and all efforts were made to minimize suffering. Review of human autopsy slides was deemed by the Seattle Children’s Institutional Review Board to not meet the federal definition of “human subjects research.”

### Study design, animal groups

Analysis of fetal brain pathology included both pregnant nonhuman primate (NHP) and human samples from 5 cohorts: (1) fetal NHP controls (*N* = 9), (2) fetal NHP – maternal ZIKV infection (*N* = 17), (3) fetal NHP – maternal FLUAV infection (*N* = 10), (4) neonatal NHP subjected to perinatal hypoxia-ischemia (*N* = 28), and (5) an autopsy cohort of human infants (*N* = 78). Groups 1–3 are part of ongoing research investigations related to the prenatal consequences of maternal FLUAV or ZIKV infection using experimentally infected healthy pregnant dams (*M. nemestrina*) with either virus or saline (sham controls) at different stages of gestation with necropsy in the third trimester (Fig. S1, Table S1). Group 4 was added to review the fetal brain histopathology from neonatal macaques, which had been subjected to brief periods of perinatal hypoxia-ischemia caused by experimental clamping of their umbilical cords, as part of a previously published study (University of Washington IACUC permit #3328-05) (Table S2) [13]. Inclusion of Group 5 enabled a similar review of fetal brain histopathology from an autopsy cohort of 78 infants who died less than one month after birth (Table S3) in a deliberate effort to identify pathology like that observed in our viral-inoculation model.

### Animal groups

An objective of our ongoing studies was to determine if the temporal course of maternal infection is associated with changes in fetal brain pathology. Therefore, we incorporated a time course into our ZIKV NHP studies to generate three study groups with different time intervals from maternal infection to delivery (Fig. S1): (1) Short (SHORT)-ZIKA (*N* = 5) inoculated in the third trimester and necropsied 2–7 days later; (2) Intermediate (INT)-ZIKA (*N* = 7) inoculated in the late second or early third trimester and necropsied 20–24 days later; and (3) Long (LONG)-ZIKA (*N* = 5) inoculated in first or second trimester and necropsied 43–97 days later. The LONG-ZIKA cohort was the basis for two prior publications significantly different in content from the current study [11, 12]. Within the FLUAV group, healthy pregnant dams were inoculated with FLUAV and Cesarean section and necropsy were performed 5 days later.

### Viral inoculations

We subcutaneously inoculated 17 healthy pregnant pigtail macaques with ZIKV (5 × 10^7^ plaque-forming units). For the SHORT-ZIKA and INT-ZIKA groups, a Brazilian ZIKV strain (Fortaleza, 2015) was used. For the LONG-ZIKA group, we inoculated a Cambodian ZIKV strain (F2213025, 2010) in 2 animals (ZIKA1, ZIKA2) and the Brazilian ZIKV strain (Fortaleza, 2015) in 3 animals (ZIKA3-ZIKA5). In the FLUAV group, we inoculated a pandemic strain of FLUAV H1N1 (A/California/07/2009) via four different routes (intratracheal, nasal, eyes, and oral) with a total inoculum of 7.4 × 10^6^ plaque-forming units into 10 healthy pregnant pigtail macaques.

### Animal care

All animals were monitored carefully for signs of illness and a battery of serological studies was performed on all dams prior to and during pregnancy to document infections with any endemic macaque pathogens; the details of the ZIKV, West Nile Virus, and dengue virus assays were described in our prior study [10]. Data was extracted from the University of Washington Primate Center Animal Research Management System database, which reflected historical testing of pathogens by the University of Washington Department of Laboratory Medicine or the WaNPRC Primate Diagnostic Services Laboratory. For many of the pathogens, testing was performed repeatedly before and during pregnancy and dams with any positive result were termed “positive” even if a later test was negative, which is the convention for test results for Monkey B Virus (Cercopithecine herpesvirus 1; CHV-1) and coccidioidomycosis (Valley Fever) (Table S4).

### Viral RNA detection assay

We have previously published ZIKV real-time quantitative polymerase chain reaction (RT-qPCR) results from brain tissue samples of LONG-ZIKA fetuses using a ZIKV prME target which revealed positive results in ZIKA1 and ZIKA2 fetal brain [10]. In this project, we used a newly designed and optimized RT-qPCR targeting the ZIKV capsid with greater sensitivity and specificity. Viral RNA load was assessed in tissues from the dam, fetus, and placenta using a ZIKV or FLUAV H1N1 virus-specific RT-qPCR assay. Fetal and maternal organs were either immersed in RNA-later immediately upon harvest or flash frozen in Tissue-Tek Optimal Cutting Temperature (OCT) compound (Cat# 4583, Sakura Finetek USA Inc, Torrance, CA, USA) and were later weighed and homogenized in either QIAzol (Cat# 79306, Qiagen, Hilden, Germany) (for brain samples) or TRIzol reagent (Cat# 15596026, Thermo Fisher Scientific, Waltham, MA, USA) (for all other tissues). Tissues were homogenized using a Precellys Evolution bead-beater apparatus with the Cryolys Evolution cooling system. (Cat# K002198-PEVO0-A.0, Bertin Technologies, Montigny-le-Bretonneux, France). Maternal or fetal plasma was separated from aseptically collected dam or cord blood in heparin tubes through centrifugation at 1,500 x g for 10 min at 4 °C.

RNA was extracted from tissues using the RNeasy mini kit (Cat# 74106, Qiagen) and from serum or plasma using the QIAamp Viral RNA Mini Kit (Cat# 52906, Qiagen) according to manufacturer instructions. 800–1000 ng of RNA was used to synthesize cDNA using the iScript select cDNA synthesis kit (Cat# 1708897BUN, Bio-Rad Laboratories, Hercules, CA, USA) according to manufacturer’s protocols for gene-specific primers or random primers. Serum or plasma was treated with *Bacteroides* Heparinase I (Cat# P0735S, New England Biolabs, Ipswich, MA, USA) after cDNA synthesis.

Viral RNA was quantified using the TaqMan Fast Advanced Master Mix (Cat# 4444556, Thermo Fisher Scientific) and an Applied Biosystems QuantStudio 3 RT-PCR system (Cat# A28567, Thermo Fisher Scientific) with primers (Table S5) that correspond to residues conserved in both the FSS13025 and Brazil Fortaleza or H1N1 genome (GenBank numbers KU955593.10, KX811222.1, NC_026433.1). The thermal cycle program for ZIKV reactions consisted of one cycle of 120 s at 95 °C, then 45 cycles of 10 s at 95 °C, 15 s at 58 °C, and then 30 s at 60 °C to capture data. FLUAV reactions consisted of a single cycle at 50 °C for 120 s followed by 120 s at 95 °C, followed by 40 cycles of 1 s at 95 °C and 20 s at 60 °C where data was captured. Samples were considered positive for detection of viral RNA if amplification curves were less than or equal to a Ct (cycle threshold) value of 40. Copy number sensitivity, as determined using a standard curve from 7 tenfold dilutions of known quantities of ZIKV or H1N1 genome, was between 3 and 20 copies/qPCR reaction.

### Plaque assay

The plaque assay was performed in two steps. The first step was to propagate live viruses from tissues or plasma by incubating plasma or tissues with a susceptible cell line. The ZIKV and FLUAV cohort samples were incubated with mosquito-derived C6/36 (Aedes albopictus clone, #CRL-1660, ATCC, Manassas, VA) or MDCK-SIAT1 (gift from Dr. Jesse Bloom) cells, respectively. The cell lines were routinely cultured in a solution of complete Dulbecco’s modified Eagle’s medium (cDMEM), comprised of DMEM (Cat# 10-013-CM, Corning, Corning, NY, USA) supplemented with 10% heat-inactivated fetal bovine serum (FBS) (Cat# SH30396.03HI, Cytiva Life Sciences, Marlborough, MA, USA), at 28 °C. The C6/36 or MDCK-SIAT1 cells were plated in 6 well-plates (1,000,000 cells/well) with 10% FBS complete media at 28 °C overnight. When the cells were about 70% confluent, the ZIKV or FLUAV plasma samples or tissue homogenates were added to each well. The tissue samples were homogenized, spun at 1,500 rpm at 4 °C, and 500 µl of the supernatant was added to each well. For plasma samples, 100 µl of plasma was added to each well. For FLUAV plasma and tissues, the supernatants from each well were recovered after three days and spun at 1,500 rpm at 4 °C for 10 min. For plasma and tissues obtained from ZIKV cohorts, the media was changed after three days and plates continued incubating for 4 additional days. Supernatants were then recovered and spun at 1,500 rpm at 4 °C for 10 min. Then, the supernatants were aliquoted and stored in -80 °C for plaque assay.

In the second step, plaque assays were performed using either Vero cells (ZIKV cohort) or MDCK-SIAT1 cells (FLUAV cohort). Approximately 1 × 10^6^ of Vero cells or MDCK-SIAT1 cells were seeded in six-well plates and incubated overnight in complete media (1x DMEM, 1X MEM Non-essential Amino Acid Solution, 10% FBS, 20 mM HEPES, 2 mM L-glutamine, and 100 U/ml penicillin and streptomycin). Serial dilutions were prepared in 1x DMEM and ~ 0.5 ml was added per well to the monolayer. The plates were then incubated for one hour (FLUAV cohort) or two hours (ZIKV cohort). After this absorption period, the viral inoculum was removed and the monolayer was washed with 1X PBS, and then 2 ml of media overlay (1.2% cellulose, 2X DMEM media) was added to each well. The plates were then incubated for 3 days (FLUAV cohort) or 5 days (ZIKV cohort) at 37 °C, until the plaques became visible, and fixed with 10% neutral buffered formalin for at least 2 h at room temperature. To visualize the plaques, the wells were stained with 0.5% crystal violet in 10% ethanol. The plaques in each well were counted. The results should be interpreted as semi-quantitative given the initial amplification step that was performed to enable detection of live virus at extremely low counts.

### Necropsy and tissue sampling

At each macaque necropsy, the fetal cerebral hemispheres, cerebellum, and brainstem were divided in the mid-sagittal plane and one-half of the brain was fixed in 4% paraformaldehyde. The fixed halves were subsequently “bread loafed” to produce 10–11 coronal slabs (each ~ 4 mm thick) of the cerebrum and 3–4 transverse sections of brainstem / cerebellum, which were embedded in paraffin. Four micron-thick histological sections from these tissue samples were used for all histological and immunohistochemical studies. From the opposite half of the brain, separate RNA later and snap frozen samples were collected from cortical gray matter, cortical white matter, deep gray matter, right cerebellum, and right brainstem. In addition, ~1mm^3^ pieces of white matter lateral to the lateral geniculate nucleus and lateral to the frontal horn of the lateral ventricle were fixed in 4% glutaraldehyde in 0.1 M sodium cacodylate buffer. The dam’s brain was handled in a similar manner. Samples from all major organs of the fetus and dam were also snap frozen and processed for histology. The placental chorioamniotic membranes, fetal meninges and fetal blood were cultured to rule out bacterial contamination of the placenta or the fetus.

### Histology, immunohistochemistry, and electron microscopy

Hematoxylin-and-eosin (H&E)-stained sections from every organ were evaluated. Except for visceral pathology observed in some of the FLUAV-inoculated dams, which will be reported separately, and the brain findings reported below, no significant consistent pathology was observed in the other dams, placenta, or any of the other fetal tissues. Coronal sections of the cerebral hemispheres were reviewed independently by two pathologists (R.K. and A.B.) with no significant discordant interpretation. Sections from many of the cerebral tissue samples were also pretreated with diastase and stained with periodic acid–Schiff–diastase stain (PASd) to highlight deposits of glycoproteins or glycolipids. Inclusion cells (ICs), as described in the results, were counted in each H&E-stained coronal section to establish the maximal number of ICs present in one coronal section from each brain.

Immunohistochemistry was performed with a Ventana Benchmark II automated immunostainer using the Optiview detection system (Ventana BenchMark Ultra; Ventana Medical Systems, Tuscon, AZ). The primary antibodies used in this study and relevant immunolabeling parameters are provided in Table S6. Briefly, we stained tissues with a battery of antibodies for IC characterization including: lysosomal-associated membrane protein 1 (LAMP1), lysosomal-associated membrane protein 2 (LAMP2), glial fibrillary acidic protein (GFAP), ionized calcium-binding adapter molecule 1 (IBA1), cathepsin S (CTSS), microtubule-associated protein 1 A/1B-light chain 3 (LC3), SRY-box 2(SOX2), SRY-box transcription factor 10 (SOX10), oligodendrocyte transcription factor 2 (OLIG2), myelin basic protein (MBP), marker of proliferation Kiel 67 (Ki67/MIB1), neurofilament, neuron-specific nuclear protein (NeuN), calretinin (CALB2), nucleoporin 62 (NUP62), caspase 3 (CASP3), and ZIKV non-structural protein 1 (NS1).

For LAMP1-, LAMP2-, and IBA-1-immunostained sections, optical densitometry was performed to quantify areas of immunoreactivity. Individual 200x fields of areas were photographed. Densitometry was performed by observers blinded to the pathogen-exposure history of each specimen. Digital Image Analysis (DIA) platform Visiopharm Integrator System (VIS; ver. 2023.01.1.13563; Visiopharm, Hørsholm, Denmark) was used to analyze the immunohistochemistry stains. Positive staining was detected by binary thresholding. The percent positive staining was calculated by comparing the area of the positive stain label to the whole tissue section area. Sections used for quantification of immunoreactive cells were counterstained with H&E. Other immunolabelled sections were counterstained with PASd to exclude or confirm colocalization of various antigens in the nuclei or cytoplasm of PASd-positive ICs. Distributions of ICs and areas of abundant or sparse LAMP1- and LAMP2-immunoreactive glial cells were mapped on drawings of coronal sections from the BrainInfo Macaque Atlas [14].

As controls for ZIKV immunohistochemistry, approximately 10 million virus-infected and uninfected Vero cells were collected from monolayers using trypsin enzymatic digestion. Cells and media were centrifuged at 800 rpm for 10–20 min and then washed with PBS. The cell pellets were then fixed with 10% neutral buffered formalin (NBF) for 24 h at room temperature. The cell pellets were then washed and resuspended with 70% ethanol at 4 °C for 24 h. Next, the cell pellets were spun for 10 min at 1,500 rpm and the ethanol was discarded and drained from the tube. The cell pellets were then added to the Epredia Cytoblock system (Cat# 7401150, Fisher Scientific) and prepared following manufacturer instructions. The lid of the Cytoblock cassette was closed and placed in 70% ethanol. The FFPE cell pellets were then embedded in paraffin and sectioned for staining and viewing.

Glutaraldehyde-fixed samples of deep white matter were washed 5 times for 5 min each time in buffer at room temperature and post fixed in buffered 2% osmium tetroxide, on ice, for 1 h. After 5 washes in distilled water and en bloc staining in 1% uranyl acetate overnight at 4 °C, the tissue was washed 5 × 5 min in water, dehydrated in ice cold 30%, 50%, 70%, and 95% ethanol, and then allowed to come to room temperature. This was followed by two changes of 100% ethanol, 2 changes of propylene oxide, and infiltration by a 1:1 mixture of propylene oxide: Epon Araldite resin for 2 h. Next, fresh Epon Araldite was exchanged with the mixture twice (2 h per change) and the tissues were embedded in Epon Araldite at 60 °C overnight. The tissues were cut to yield 80 nm sections that were stained for 2 min in Reynold’s lead citrate and imaged with a transmission electron microscope (JEOL USA, Peabody, MA, USA) at 80 KV.

### Autofluorescence

Autofluorescence was assessed in 5-µm-thick paraffin sections from IC-rich brain tissue along with sections of lipofuscin-containing macaque cerebellum as a positive control. Sections were deparaffinized and incubated for 10 min in a dilute solution of 4′,6-diamidino-2-phenylinverdole (DAPI; 5 ul in 50 ml water), rinsed, and coverslipped in water. Photomicrographs of autofluorescence (green– excitation 467–498 nm and red – excitation 542–582 nm) and DAPI (excitation 352–402 nm) were taken and then the coverslip was removed and PASd staining was performed. The same microscopic fields were identified and rephotographed under brightfield optics.

### Review of brain slides from human cases and other models of brain injury

A review was conducted of H&E-stained coronal sections from the brains of neonatal macaques, which had been subjected to brief periods of perinatal hypoxia-ischemia caused by experimental clamping of their umbilical cords, as part of a previously published study (University of Washington IACUC permit #3328-05) [13]. Details concerning the experimental manipulations and some of the findings in these animals are provided in Table S2. A similar review of H&E-stained brain slides was performed in an autopsy cohort of 78 infants, who died less than one month after birth (Table S3). Paraffin sections from the same tissue blocks were prepared from a subset of these patients and either stained with PASd or immunohistochemically, as described above. These cohorts were included in a deliberate effort to identify ICs like those observed in our pregnant NHP viral inoculation models.

### Statistics

Fetal inclusion cells were analyzed as a continuous variable and binary variable using different thresholds. When fetal inclusion cell positivity was considered a binary variable, we used the Fisher exact test or the Kruskal-Wallis test. When the analysis was performed as a continuous variable, we used the Spearman rank-order correlation test. A p-value less than 0.05 was considered significant.

## Results

### Inclusion cells (ICs)

**Fig. 1:**
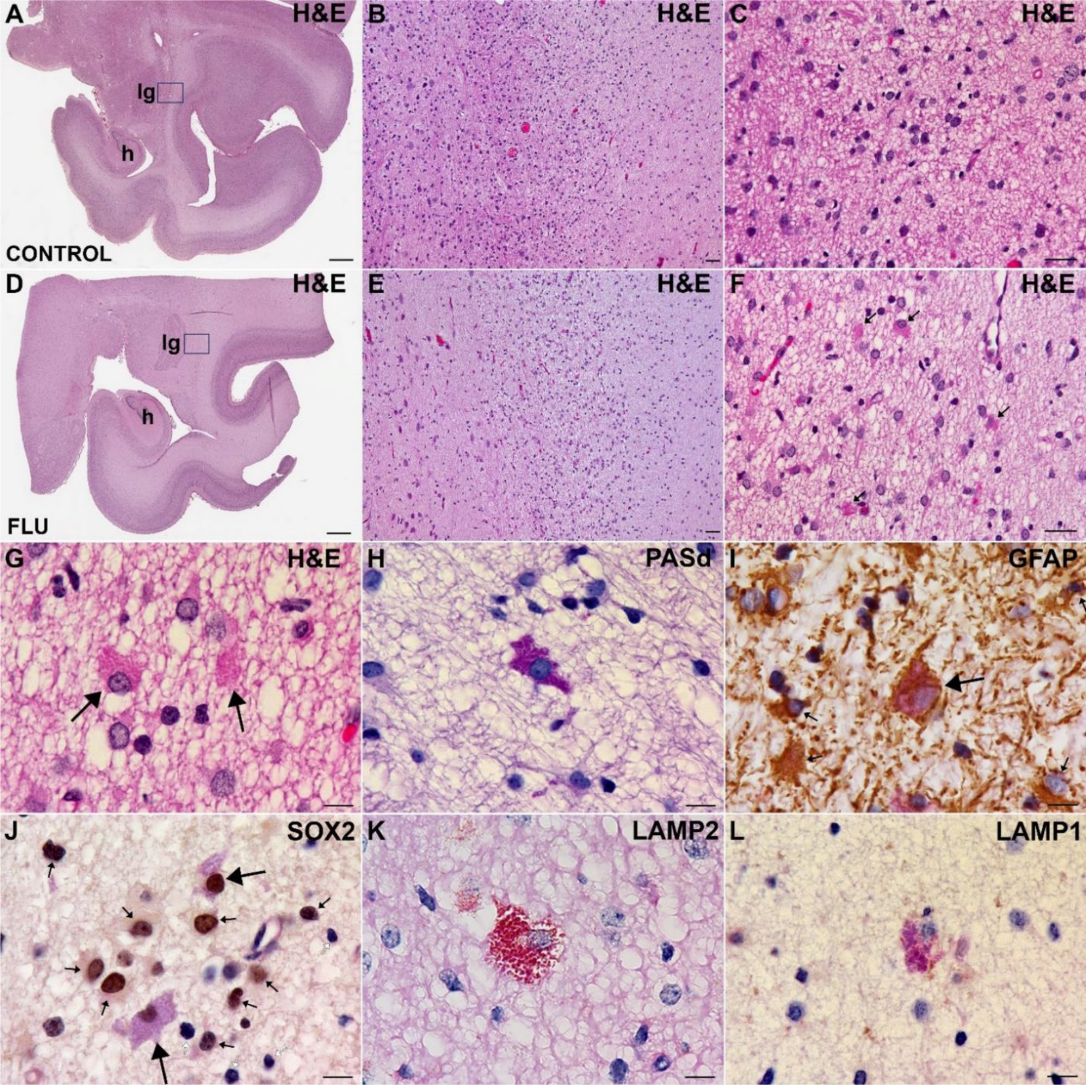
Histological and Immunohistochemical Properties of Inclusion Cells. (**A**-**C**) The deep white matter adjacent to the lateral geniculate nucleus (lg) of a control fetus contains astrocytes with inconspicuous agranular eosinophilic cytoplasm. (**D**-**G**) In contrast, deep white matter from the same location in a fetus exposed to influenza virus (FLUAV) contains multiple astrocytes with abundant cytoplasm filled with granular eosinophilic inclusions (arrows). (**H**) Diastase-resistant, intense magenta staining of the granular inclusions is seen with periodic-acid-Schiff stain, which colocalizes in the same cells with brown immunolabelling of the astrocyte markers GFAP (I, arrow) and SOX2 (J, arrows) and the lysosomal markers LAMP2 (**K**) and LAMP1 (**L**). Abbreviations: H&E, hematoxylin & eosin; h, hippocampus. Scale bars: A, 1.5 mm; B, 100 μm; C, 25 μm; D, 1.5 mm; E, 100 μm; F, 25 μm; H-L, 10 μm

A retrospective case series of neurohistopathology was performed on a series of late-gestation pigtail macaque fetuses from 27 virus-inoculated dams (10 FLUAV; 17 ZIKV) and 9 sham-inoculated controls with varying time intervals between maternal inoculation and necropsy (Fig. S1, Table S1). The microscopic evaluation of the fetal brains revealed a variably dense population of atypical glial cells in the deep white matter of a subset of the fetuses, which we called “inclusion cells” (ICs) and prompted further investigation. The atypical ICs had round nuclei, irregular perikaryal membrane contours, and abundant perinuclear cytoplasm (Fig. 1). The distinctive feature of these cells was the presence of numerous, closely apposed, eosinophilic granular inclusions, which appeared to fill the cytoplasm surrounding the nucleus. The granular cytoplasmic inclusions in ICs stained intensely with PASd, which highlights glycosylated proteins and lipids (Fig. 1H).

### Brain tissue architecture

Gross pathology of the fetuses revealed no obvious congenital malformations, and fetal organs showed no consistent macroscopic pathology. The external and cut surfaces of the brains, brainstems and spinal cords of all fetuses appeared grossly normal with appropriate brain weights for age except ZIKA1, a LONG-ZIKA animal in which white matter hypoplasia was evident grossly and by prenatal imaging, as described previously [11]. H&E-stained coronal and transverse sections revealed normal brain architecture with exceptions in the LONG-ZIKA group [10, 11]; ependymal loss and periventricular gliosis was observed in the occipital horns of 4 of the 5 fetuses in the LONG-ZIKA group, as reported previously [10]. These changes were not identified in fetuses in the SHORT-ZIKA, INT-ZIKA, FLUAV, or in any of the controls. Maternal pathology was largely restricted to the lungs of some FLUAV-inoculated dams and will be reported separately. Except for two of the LONG-ZIKA fetuses [10], there were no consistent macroscopic pathologic features or marked changes in brain architecture that characterized most fetal brain samples or any of the brains with ICs.

### Immunohistochemical characterization of ICs

**Fig. 2:**
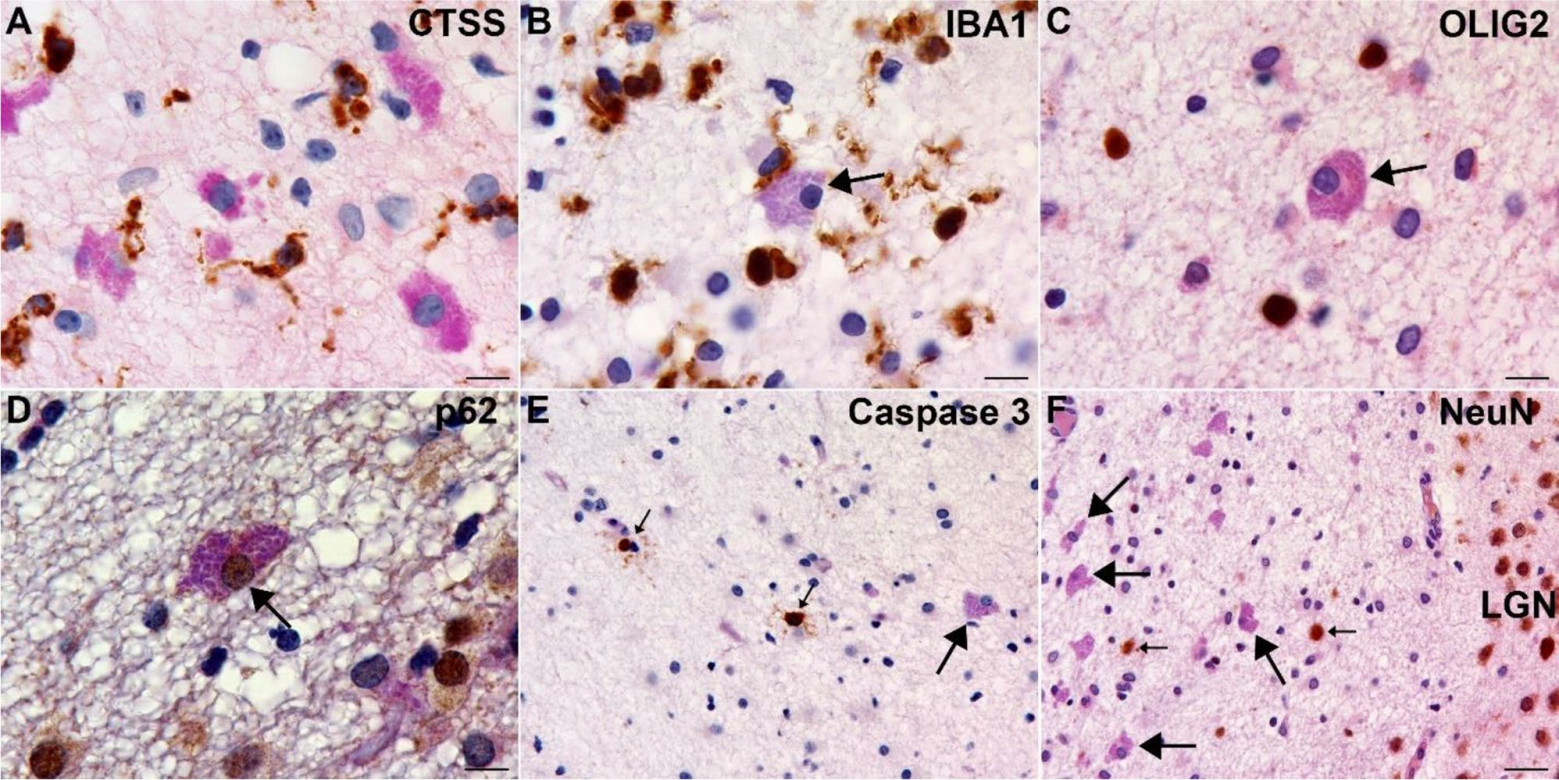
Additional Immunohistochemical Properties of Inclusion Cells. In PASd, counterstained sections, no labeling of inclusion cells is observed with the microglial markers cathepsin S (CTSS, **A**) or IBA-1 (**B**), the oligodendroglia marker (OLIG2, **C**), or the neuronal marker (NeuN, F). Small arrows in F indicate immunoreactive white matter neurons. (**D**) Inclusion cells, as well as neighboring cells express the autophagy-associated p62 protein. (**E**) Caspase 3 immunoreactivity was not detected in inclusion cells but was present in rare small cells (small arrows) in the background with no subjective increase in inclusion cell-rich foci or elsewhere in the white matter of fetuses with inclusions and/or viral exposure. Large arrows, inclusion cells. Scale bars: A-D, 10 μm; E-F, 25 μm

A battery of immunohistochemical studies was performed to further characterize ICs (Figs. 1 and 2). Immunolabeling of the ICs was consistently observed with antibodies targeting the astrocyte antigens, GFAP and SOX2 (Fig. 1I and J). Labeling with GFAP did not show enhanced labeling, as is observed frequently with some reactive astrocyte responses to injury (Fig. S2). ICs also stained with antibodies targeting lysosomal antigens (LAMP1 and LAMP2, Fig. 1K and L) and the autophagosomal antigen, p62 (Fig. 2D). Antibodies specific for microglia (IBA-1, CTSS), oligodendroglia (SOX10, OLIG2) or neurons (NeuN, CALB2) highlighted appropriate cells in the background, but did not recognize ICs (Fig. 2, CALB2 data not shown).

In representative sections from each group, Caspase 3 immunohistochemistry only labeled rare cells in the vicinity of ICs, most of which had nuclear features identical to the apoptotic bodies identified in H&E-stained sections. Similarly, there was sparse Ki67 immunoreactivity in the vicinity of the ICs (not shown) of each group and neither Caspase 3 (Fig. 2E) nor Ki67 highlighted ICs. Apart from the presence of ICs, the microscopic anatomy and cellular background within the fetal brains was not appreciably different in the brains with numerous versus few ICs. An identical histological evaluation was performed on the adult dams of each fetus and no ICs were identified in any of these brains.

### Ultrastructural appearance of inclusion cells

**Fig. 3:**
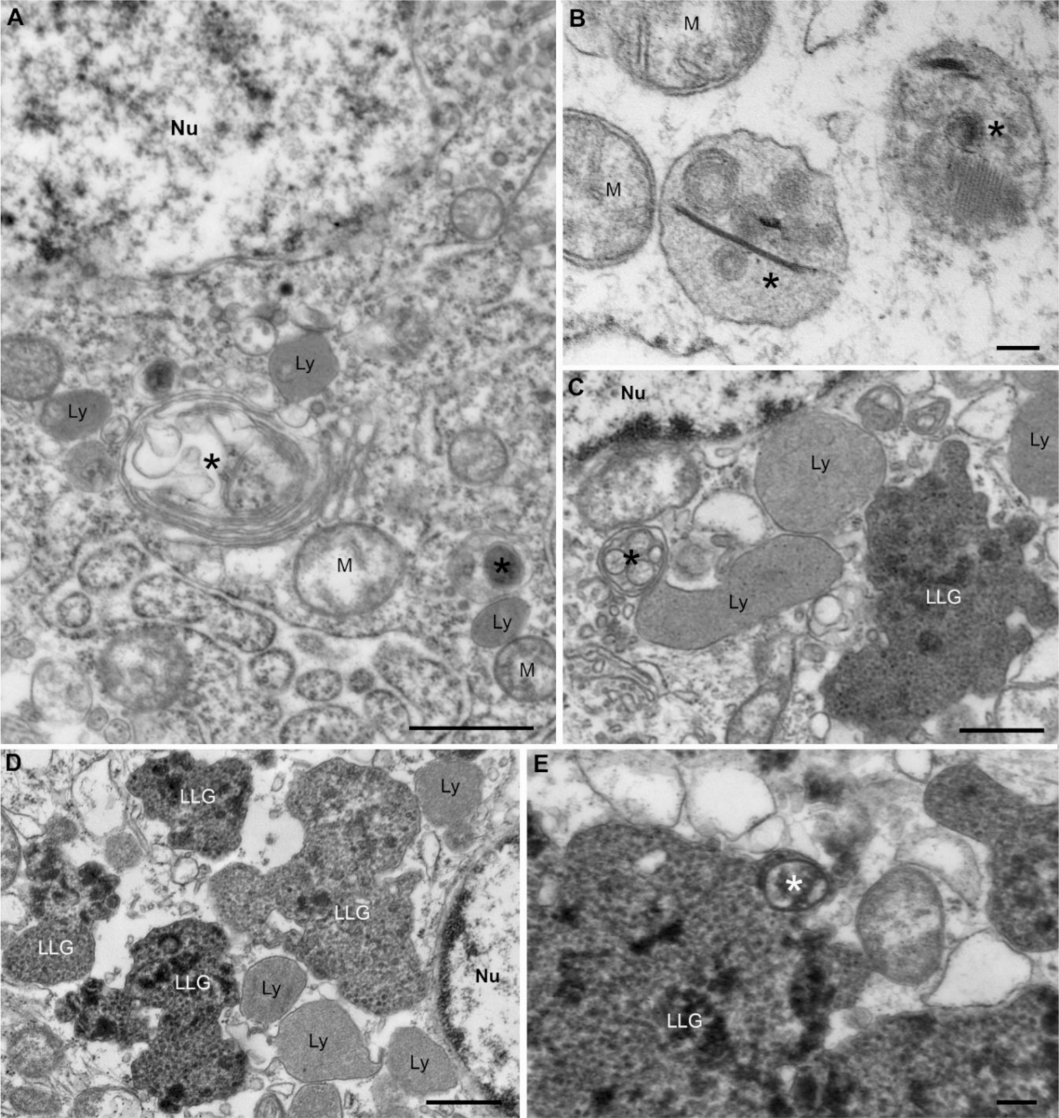
Ultrastructural Features of Inclusion Cells and Neighboring Astrocytes. (**A**-**B**) Astrocytes neighboring inclusion cells occasionally showed evidence of early autophagosome formation including membrane-bound degenerating organelles (asterisks) with adjacent lysosomes (Ly) and mitochondria (M). (**C**-**E**) Inclusion cells contained similar structures (asterisks), abundant lysosomes (Ly) and irregularly shaped lipofuscin-like granules (LLG), the distribution and sizes of which corresponded well with the PASd-positive inclusions observed by light microscopy. LLGs had heterogenous electron-dense compositions including granular and membranous areas. Nu, nucleus. Scale bars: A, 600 nm; B, 100 nm; C, 600 nm; D, 600 nm; E, 200 nm

Electron microscopy was performed to investigate the ultrastructural appearance of ICs, which supported that they were astrocytes with abundant electron-dense, membrane-bound inclusions in their cytoplasms (Fig. 3). Their cytoplasmic inclusions closely resembled lipofuscin, a membrane-bound complex mixture of oxidized protein and lipid degradation residues, which can represent a late stage in autophagy [15–17]. However, unlike lipofuscin, these cytoplasmic granules were not autofluorescent (Fig. S3). Consistent with the inclusions representing some type of lysosomal or autophagosomal process, abundant lysosomes were present in the cytoplasm of ICs as well as autophagosomes, which contained degenerating organelles, although the latter were relatively sparse. Occasional neighboring astrocytes contained prominent lysosomes without autophagosomes.

### Spatial distribution of ICs

**Fig. 4:**
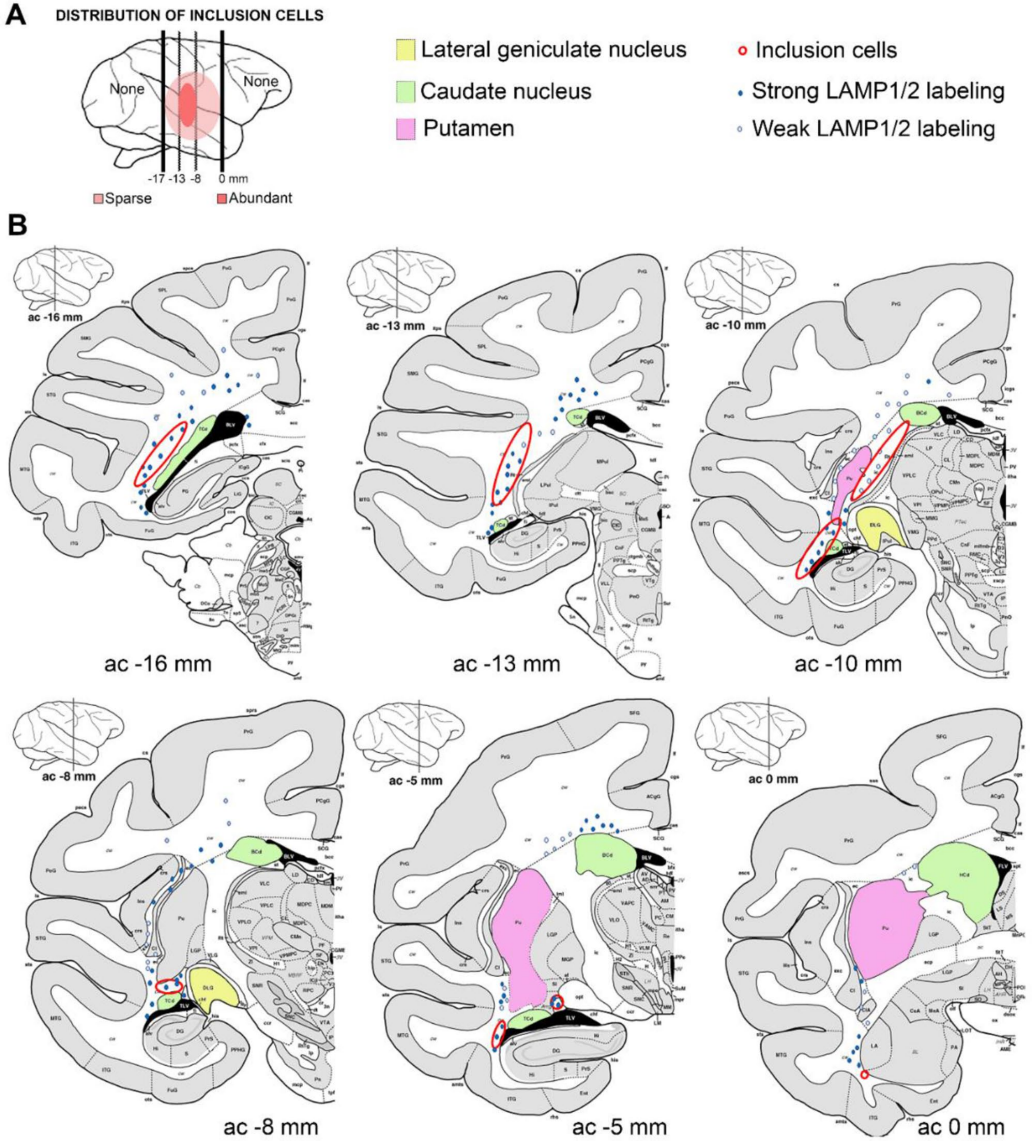
Distribution of Inclusion Cells. (**A**) Inclusion cells were only observed in the deep white matter in central portions of the cerebrum, predominantly in sections between − 8 mm and − 13 mm in the macaque brain atlas (BrainInfo, 1991-present, National Primate Research Center, University of Washington, http://www.braininfo.org). This work is licensed under a Creative Commons Attribution 3.0 Unported License. (**B**) Annotated diagrams of coronal sections from the atlas demonstrate the overlap between the locations in which inclusion cells are found (red outlined areas) and weakly (open circles) or strongly (filled circles) LAMP1/2-positive glial cells in the brains of fetuses with inclusion cells. ac, atlas coordinate

Next, we determined the spatial distribution and density of ICs across different cohorts. First, we defined an IC region (ICR), which corresponded to portions of the white matter between coronal levels 0 mm and − 17 mm in the BrainInfo Macaque Atlas [14]; no ICs were identified outside these coronal levels in any of the fetuses. Our review of routine coronal sections (~ 4 mm intervals) revealed that ICs were most prevalent in the deep white matter lateral and ventral to the thalamus in a fronto-occipital distribution; this distribution extended from the stria terminalis, just occipital to the optic chiasm, to the coronal level of the posterior pulvinar (Fig. 4). The ICR extended superiorly in the white matter on the lateral aspect of the putamen/claustrum and pulvinar, but ICs were generally not present superior to the level of the mid thalamus.

**Fig. 5:**
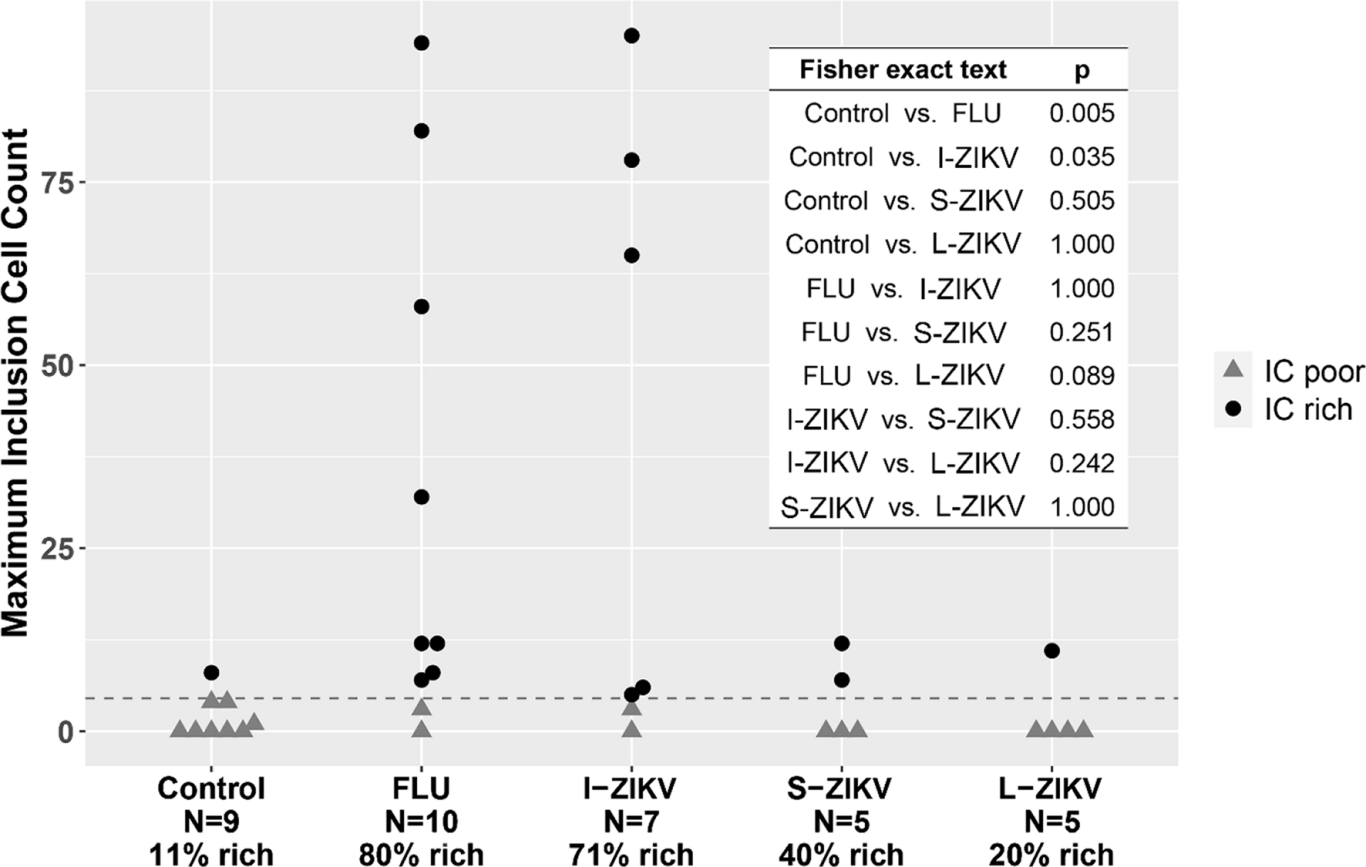
Maximal inclusion cell number in IC-Rich versus IC-Poor groups. The figure shows the maximal number of inclusion cells identified in any coronal section from each of the fetuses. The data are grouped according to pathogen exposure and, for Zika fetuses, the interval between maternal inoculation and necropsy. Different threshold levels were trialed to identify the lowest threshold to separate “IC-rich” (points above line) from “IC-poor” (points below line) that yielded significant differences (*p* < 0.05 with Fisher exact test) between the groups. At a cut-off value of 4.5 inclusion cells (dashed line), a significantly greater relative number IC-rich to IC-poor fetuses is present in the FLUAV and INT-ZIKA (I-ZIKV) groups as compared to controls or the SHORT-ZIKA (S-ZIKV) or LONG-ZIKA (L-ZIKV) groups (table in inset). N, total number of animals in each group; percentages shown reflect fraction of IC-rich fetuses in each group

The number of ICs identified in an individual coronal section from a single brain ranged from 0 to 95 (Fig. 5; Table 1). A natural break was observed at a threshold of 5 ICs per coronal section in which only 11% (1/9) of controls exceeded that cutoff versus 59% (16/27) of the pathogen (FLUAV + ZIKV) group. Notably, ICs were rare in fetal brains from all controls, SHORT- and LONG-ZIKA cohorts, which established a group that we called “IC-poor”. In contrast, ICs (≥ 5 per coronal section) were significantly more prevalent in the FLUAV and INT-ZIKA cohorts versus controls (*p* < 0.005 and *p* = 0.04, respectively), which we referred to as an “IC-rich” cohort). In all fetuses, the maximal density of ICs was centered in a site ventral to the caudal putamen and lateral to the lateral geniculate nucleus extending rostrally to white matter between the optic tract and thalamus, including the subventricular zone on the lateral aspect of the temporal ventricular horn. The center of the ICR was in the deep white matter, where the density of astrocytes, microglia, and oligodendroglia is normally greater than in subcortical white matter (Fig. 4). ICs were not present in non-ICR deep white matter (e.g. deep white matter superior and lateral to the body of the caudate nucleus) where astrocytes and other cells lacked ultrastructural evidence of autophagy or other atypia.

**Table 1:** Inclusion cell-poor and inclusion cell-rich groups

Pathogen Group	Inclusion cell category				*p* value* (vs. Controls)
	POOR ≤ 4		RICH 5+		
	*N*	Max count per fetus	*N*	Max count per fetus	
**Control**	8	0, 0, 0, 0, 0, 1, 4, 4	1	8	
**FLU**	2	0, 3	8	7, 8, 12, 12, 32, 58, 82, 94	0.005
**INT-ZIKV**	2	0, 3	5	5, 6, 65, 78, 95	0.035
**S-ZIKV**	3	0, 0, 0	2	7, 12	0.505
**L-ZIKV**	4	0, 0, 0, 0	1	11	1

* Within each group, the commas separate the maximum number of inclusion cells identified in a single H&E-stained coronal section from the brain of each fetus

IC-rich fetuses were generally younger at the time of delivery than IC-poor fetuses (Fig. S4). All but one (16/17, 94%) of the IC-rich fetuses were delivered at a gestational age of less than 150 days versus 10/19 (53%) of the IC-poor fetuses. This difference was significant but is confounded by the fact that age of delivery was influenced by the day of inoculation and interval to delivery (*p* < 0.0001). Per protocol, FLUAV inoculations were performed between gestational days 124 and 138 with delivery 5 days later. The FLUAV cohort had not only the earliest inoculations, but the greatest systemic disease and highest inclusion cell counts. In contrast, LONG-ZIKA fetuses predominated in the oldest gestational age range at delivery and had no systemic disease and no or only rare ICs. Interestingly, the only IC-rich LONG-ZIKA was the youngest, which delivered at 142 days.

**Fig. 6:**
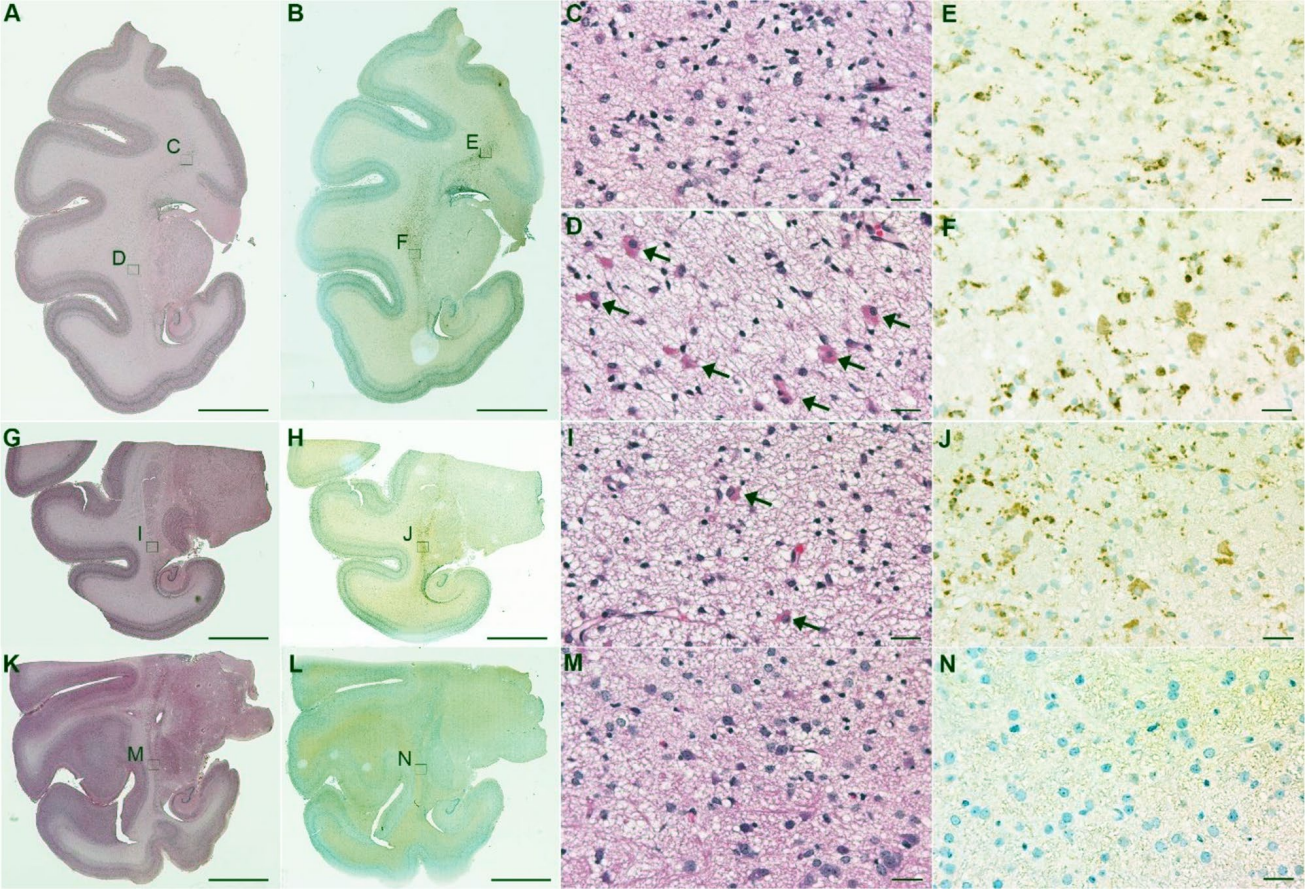
Representative Images of LAMP1 Immunohistochemical Labeling in ICR of IC-rich and IC-poor Brains. Neighboring H&E-stained (**A**) and LAMP1-immunostained (**B**) coronal sections from the IC-rich brain of a FLUAV fetus are annotated to show the locations of areas of deep white matter shown at higher magnification in **C**-**E**. Abundant inclusion cells are present in the peri-thalamic deep white matter (**D**) but absent in dorsal deep white matter dorsolateral to the corpus callosum (**C**). Intense LAMP1 expression is observed in both sites (**E**, **F**). A more occipital section from the same brain (**G**, **H**) contains fewer inclusion cells in the inclusion cell region (**I**) but similar abundant LAMP1 immunoreactive cells (**J**). (**K**, **L**) In contrast, sections from comparable areas in an IC-poor control brain are devoid of inclusion cells (**M**) and LAMP1 immunoreactivity (**N**). Scale bars: A, B, G, H, K, L, 5 mm; C, D, E, F, I, J, M, N, 25 μm

**Fig. 7:**
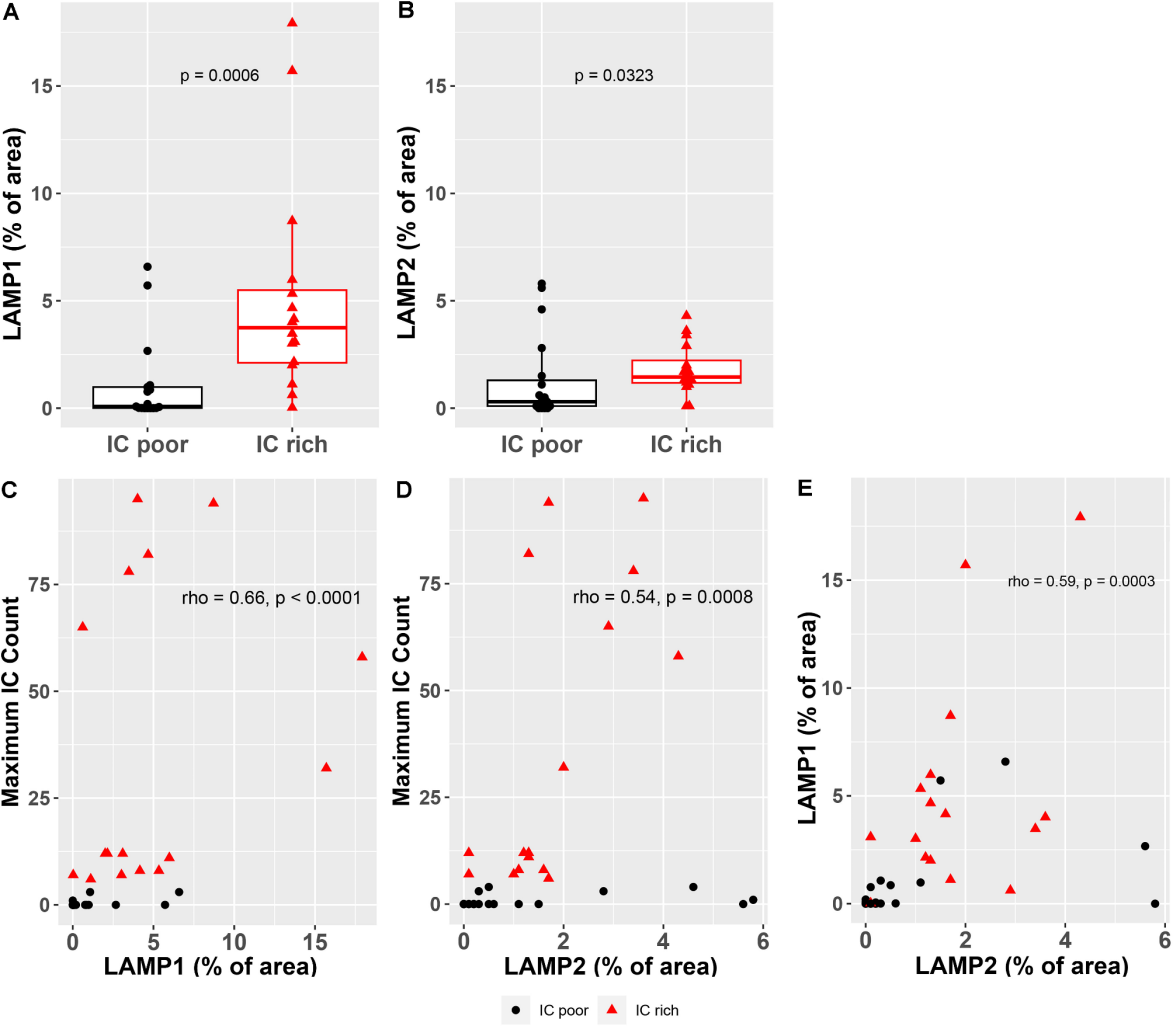
Optical Densitometry Quantification of LAMP1 and LAMP2 Immunoreactivity in the Inclusion Cell Region of Fetal Brains. (**A**, **B**) LAMP1 (**A**) or LAMP2 (**B**) positive staining, as a percentage of histological section area, was significantly greater for the cohort of IC-rich versus IC-poor brains. (**C**, **D**) Positive correlations were observed between the fractional amount of LAMP1 (**C**) and LAMP2 (**D**) immunopositivity and the maximal IC count obtained from individual fetal brains. (**E**) LAMP1 and LAMP2 immunostaining from sections of the same brain, as a percentage of histological area, correlated positively with each other

In coronal sections, astrocyte immunoreactivity for LAMP1 and LAMP2 extended beyond the ICR into deep white matter adjacent to the superior thalamus and frontal horn of the lateral ventricle. LAMP1 and LAMP2 labeling in the ICR was absent to diffuse with strong cytoplasmic staining including some larger cells which resembled ICs in IC-rich fetuses (Figs. 1K and L and 6, Fig. S5). Optical densitometry confirmed a significant increase in the amount of LAMP1 and LAMP2 immunoreactivity in the ICR of IC-rich versus IC-poor brains (*p* < 0.05, Fig. 7A, B). There was a significant positive correlation between the prevalence of inclusion cells and LAMP1 or LAMP2 immunoreactivity, although there was considerable overlap between the two groups, including some IC-poor fetal brains with relatively abundant LAMP1 and/or LAMP2 labeling (*p* < 0.01, Fig. 7C, D). The correlation between LAMP1 and LAMP2 densitometry for individual brains were good, indicating that both markers of lysosomal activation tended to up-regulate in parallel with each other (rho = 0.59; *p* = 0.0003, Fig. 7E). Neither GFAP nor SOX2 staining showed an obvious increase or decrease in the density of immunoreactive cells in the ICR of the pathogen-inoculated group versus controls or the IC-rich versus IC-poor brains (data not shown).

ICs were concentrated in the ICR but did not form cohesive or confluent groups. Instead, they were intermixed with surrounding neuropil, including oligodendroglia, microglia, and astrocytes. In this area and other non-ICR parts of the deep white matter, occasional apoptotic bodies (fragmented pyknotic nuclei) and axonal spheroids were identified, but there was no obvious difference in the frequency of these findings in the IC-bearing brains (data not shown). Vessels in the region appeared patent without necrosis, or evidence of remote or recent hemorrhage was identified. No lymphocytic, neutrophilic, or eosinophilic inflammation was present in any of the brain sections. Only white matter contained ICs, except in one FLUAV and one control in which discrete densely packed microscopic nests of ICs and associated microglia were present in the medial thalamus (Fig. S6).

### Microglial activation in IC-rich white matter

**Fig. 8:**
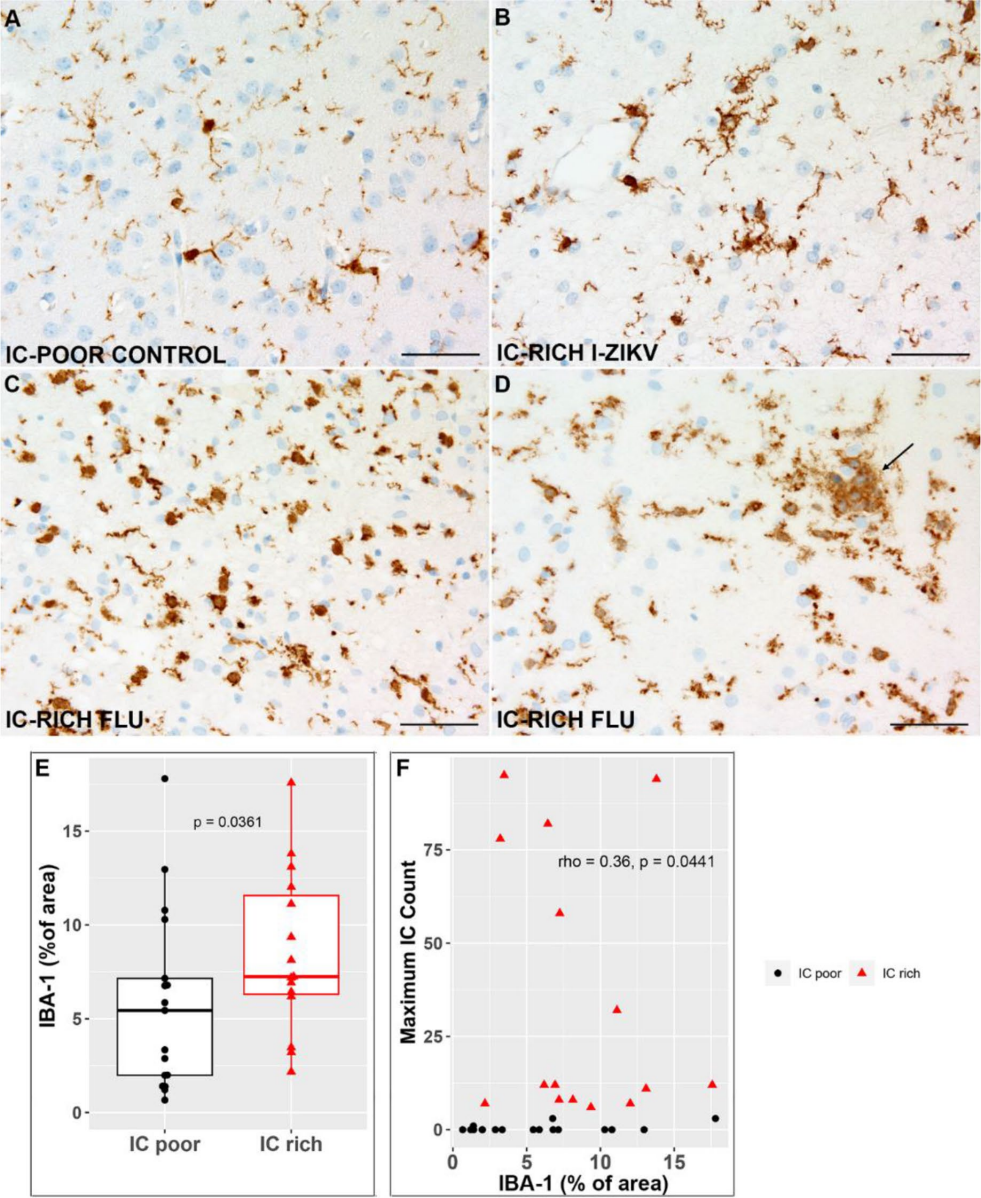
Microglial Activation in the Inclusion Cell Region of Fetal Brains. Representative images of IBA1 immunohistochemistry from the ICR of IC-poor control (**A**) and IC-rich (**B**-**D**) fetuses. (**A**) IBA1 labeling in small round cell bodies and delicate branching processes of microglial cells in this IC-poor control. (**B**-**D**) Variable numbers of larger microglial cells with short or absent processes are present in the ICR of three different IC-rich brains. Occasionally, these “amoeboid” microglia form small microaggregates (arrow in D). (**E**, **F**) IBA-1 positive staining, as a percentage of histological section area, was significantly greater for the cohort of IC-rich versus IC-poor brains (**E**), but no correlation was observed between the fractional amount of IBA-1 immunoreactivity and the maximal IC count from the same brain (**F**). Scale bars = 50 μm

Although ICs did not express the microglial markers IBA1 and CTSS, microglial cells expressing both markers were prevalent in the background (Fig. 8A-D). Optical densitometry performed on comparable microscopic fields from the ICR showed significant differences between groups in the fractional area of IBA-1 immunoreactivity with significantly more immunoreactivity in FLUAV and INT-ZIKA, as opposed to the other groups (*p* < 0.05, Fig. 8E). However, unlike LAMP1 and LAMP2, immunostaining for IBA-1 did not correlate well with the prevalence of ICs in brain sections (Fig. 8F–IBA1 graphs). In addition, the shapes and clustering of many of the IBA-1-positive cells in the ICRs with ICs were subjectively different from ICRs of brains without ICs. Specifically, a subset of IBA-1 positive cells in the ICRs of IC-rich brains had activated features including larger “amoeboid” cell bodies with thicker, shorter, and less branched appearing cell processes (Fig. 8A-D) [18]. In the ICR of some IC-rich brains, rare aggregated microglial cells were present (Fig. 8D); something never observed in the IC-poor brains. CTSS-positive cells showed similar cytological features (data not shown). CD163, a putative marker of activated anti-inflammatory microglia [18], was not expressed by these cells, in contrast with perivascular and meningeal macrophages in the same tissue sections (data not shown).

### Absence of other signs of white matter injury in inclusion cell-rich fetal brains

**Fig. 9:**
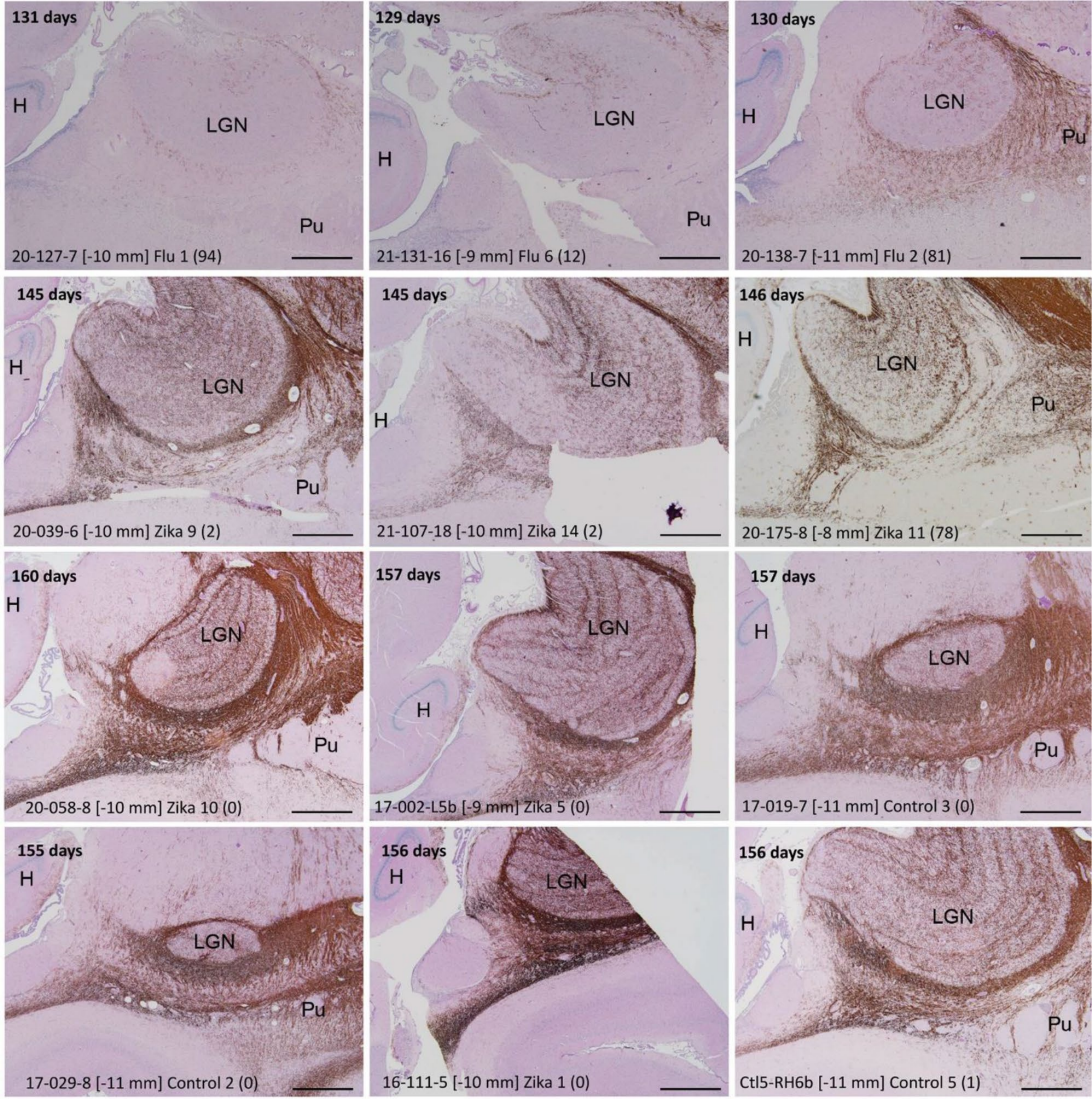
Myelin Basic Protein Immunolabeling in Inclusion Cell-Rich Region. MBP-immunostained sections (with PASd counterstain) from a large subset of fetuses included in this study span a 27-day-time period (gestational ages in upper left corner of each panel) in the third trimester when fetuses were delivered. The fields shown encompass the area in the deep white matter, lateral to the lateral geniculate nucleus (LGN) and ventral to the putamen (Pu), where inclusion cells are most prevalent. Data in the lower left corner of each image includes fetus study identifier, corresponding coronal level in BrainInfo Macaque Atlas [14], and maximal number of inclusion cells identified in that fetus’ brain sections. H, hippocampus. Scale bars: 1 mm

The ages of fetuses at delivery in this study spanned an approximate 4-week period in the third trimester and corresponded to a period of active myelination of deep cerebral white matter. Previously we reported indices of white matter injury in the parietal cortex LONG-ZIKA fetuses of dams inoculated with virus during the first trimester [12]. In this study, we focused on the ICR of the SHORT-ZIKA, INT-ZIKA, FLUAV, and control cohorts. As expected, MBP-immunostained sections demonstrated progressive age-related increases in the intensity and area of white matter immunoreactivity (Fig. 9). However, no obvious deficiency or alteration in MBP immunolabeling was observed in any of the virus inoculated cohorts versus controls or in fetuses with versus without inclusion cells, even in the sites where inclusion cells were most abundant. Similarly, no consistent differences were observed in neurofilament immunostaining (not shown) or the ultrastructure of axons with and without myelin sheaths (Fig. S7).

### Viral detection and pathogen seropositivity

Next, we determined if viral detection in the dam, placenta or fetus was associated with ICs. In the ZIKA cohorts, maternal viremia was demonstrated two days after inoculation in all maternal plasma or serum samples by RT-qPCR analysis and/or plaque assay except ZIKA1 and ZIKA3 (data not shown). In the FLUAV cohort, all maternal lungs were positive for FLUAV at necropsy by RT-qPCR or plaque assay. FLUAV was detected in the placentas of FLUAV cohort fetuses, and ZIKV was detected in some placental tissues, liver, and plasma of ZIKV fetuses (Table S7). The only central nervous system samples from which virus was detected were the cerebrum, spinal cord, and meninges of ZIKA1 (LONG-ZIKA) and meninges of ZIKA16 (SHORT-ZIKA). With one exception, virus was not detected by RT-qPCR in fetal cerebral samples at the time of necropsy, nor was ZIKV viral antigen detected immunohistochemically in fetal brain sections (Fig. S8), including the single LONG-ZIKA fetus with RT-qPCR-positive cerebrum, meninges and spinal cord tissue (Table S8). Further, seropositivity for various endemic pathogens to nonhuman primates did not correlate with IC-poor vs. IC-rich status (Table S4). We also cultured swabs taken from the placental chorioamniotic membranes and fetal meninges to rule out bacterial infection, which were negative in all cases. No significant associations were found between IC-rich and IC-poor cohorts for the frequency of viral RNA positivity in either fetal tissues, placental tissues, or both fetal and placental tissues. In summary, there was no defining profile of viral detection in the fetus or pathogen seropositivity in the dam that was associated with fetal ICs.

### Absence of inclusion cells in brains with perinatal hypoxic-ischemic injury

As ICs may be an index of prenatal white matter injury from maternal viral inoculation, we hypothesized that ICs may also be prevalent in the context of other sources of deep white matter damage. Although hypoxia-ischemia is a frequent source of fetal and perinatal white matter injury, ICs have not been described in human brains or non-human primate models with hypoxic-ischemic encephalopathy. H&E- and PASd-stained sections from a series of 78 human autopsied neonates, including many born prematurely and many with clinical and / or pathological evidence of hypoxic-ischemic encephalopathy were examined (Table S3). Sections from the central ICR, immediately lateral to the lateral geniculate nucleus, were available from 20 of the infants. No ICs were identified. Similarly, no ICs were identified in the ICR or other brain sections on retrospective review of a series of 21 pigtail macaques which were exposed to experimental perinatal hypoxia (umbilical cord clamping) (Table S2) as part of a previously published study [13].

## Discussion

In this study, we report deep cerebral white matter alterations in fetal brains after inoculation of pregnant macaques with two different viruses during the third trimester of gestation. The findings include dramatic changes in the morphology and immunohistochemical properties of microglia and astrocytes, which are heralded by cytoplasmic inclusions and lysosomal activation in a subset of astrocytes that we have termed “inclusion cells” (ICs). Ultrastructural and immunohistochemical examination of ICs indicates that the cytoplasmic inclusions share some properties with lipofuscin and are derived, at least in part, from autophagosomes. To our knowledge, ICs have not been previously described in NHP or other animal models of prenatal white matter injury. In our macaque fetuses, the presence of these cells correlated with signs of more global microglial activation and astrocyte reactivity in the deep white matter, which could be demonstrated immunohistochemically, but was otherwise inapparent in routine H&E-stained sections. ICs and related glial changes were prevalent at relatively short time intervals (5–21 days) after FLUAV or ZIKV inoculation, were exceedingly rare among LONG-ZIKA fetuses with long intervals between inoculation and necropsy, and were not associated with histological, immunohistochemical or molecular evidence for viral infection of the fetus’ brain or other tissues. Overall, the transient and temporal nature of ICs and related glial changes are likely to represent a consequence of a maternal-placental-fetal inflammatory response, which may be a marker for the development of neurocognitive or neuropsychiatric disease in humans.

### ICs correlate with astrocyte and microglial reactivity

In our experimental models, recognition of ICs in H&E-stained sections provided the first hint of altered glial properties in the deep white matter of brains of fetuses after viral inoculation of pregnant NHPs. Cells with ICs represent a small subset of a larger population of astrocytes expressing lysosomal markers LAMP1 and LAMP2, which occupy a broader region of deep white matter adjacent to parts of the thalamus and adjacent basal ganglia. The deep white matter of IC-rich animals also contained an excess of microglial cells with reactive cytological features, although neither microglial activation nor other indications of astrocyte reactivity was apparent in H&E-stained sections; these were only resolved immunohistochemically. In histological sections from the same brains, a positive correlation was observed between the percentage of LAMP1 and LAMP2 immunolabelling, although the latter was consistently less overall and more concentrated in IC-rich foci. The difference may reflect in part, different roles played by the two lysosomal proteins, LAMP2 being particularly important for autophagy [19, 20].

It seems clear from the ultrastructural appearance and immunohistochemical properties of ICs that their characteristic cytoplasmic inclusions are part of a lysosomal degradation pathway. At least in part, inclusion bodies appear to result from autophagy and resemble some published images of late-stage autophagolysosomes [21–23] or multivesicular bodies [24]. Although recent in vitro study of human fibroblasts suggests that oxidative stress promotes autophagy and leads to the formation of lipofuscin granules [25] and the electron microscopic features of most of the membrane-bound inclusions in ICs resemble lipofuscin, unlike the latter they do not exhibit autofluorescence. Regardless, some type of lysosome-mediated degradation of endolysosomal cellular debris seems most likely, and autophagy is probably at least partially involved. Although astrocyte phagocytosis, as opposed to autophagy, has been described in some settings of white matter injury [26, 27], we did not observe ingestion of cellular debris in our electron microscopic studies.

Some level of autophagy is ongoing in most cells, and excessive or arrested autophagy is a common cellular response to many forms of cellular stress [28]. For example, neuronal autophagy in the ventrolateral thalamus or asphyxiated human newborns and rats has been well documented [29]. Autophagy in astrocytes has also been linked to neurodegenerative diseases and regarded as a potentially neuroprotective process, impairment of which may contributed to brain pathology [30]. Astrocytes respond to many different microenvironmental signals (e.g., interleukin-6) to adopt pro- or anti-inflammatory reactive states [31–33] and in multiple biological contexts, inflammatory cytokines have been found to regulate autophagy [34]. For example, circulating IL-6 levels are elevated in cancer patients, including those with glioblastoma, in whom it appears to promote autophagy and chemotherapy resistance in malignant glial cells [35–37]. It is tempting to speculate that, in our NHP models, virus inoculation or other maternal stressors lead to cytokine changes or other humoral factors that promote autophagy, IC formation, and microglial activation in cerebral white matter.

### ICs are most prevalent in white matter tract “crossroads”

In our models, ICs were concentrated in the deep white matter ventrolateral to the thalamus, found less often in adjacent deep white matter immediately frontal or caudal to this region, and very rarely situated as dense aggregates in thalamic gray matter. White matter in the intermediate zone of the developing primate fetal brain is known to be vulnerable to a variety of sources of injury especially hypoxic-ischemic or infectious etiologies [4, 6, 38]. In the early and middle third trimester, when viral inoculation was performed in our study, maturation of the macaque brain is accelerated in comparison with humans [39, 40] and represents a stage in which major periventricular “crossroads” in the deep white matter intermediate zone are established and begin to myelinate [41]. Kostovic and colleagues recognize six distinct periventricular crossroads (C1-C6), of which C1 and C4 correspond to sites where ICs and associated glial changes concentrate. All these crossroads undergo dynamic changes in cellularity and extracellular matrix composition during the third trimester and each crossroad is vulnerable to fetal hypoxic-ischemic or inflammatory injury [38]. C4 is located at the exit of the retroventricular portion of the internal capsule, where we most consistently observed ICs and intense LAMP 1/2 and IBA1 immunoreactivity after viral inoculation. However, in most affected fetuses, the immunohistochemical alterations, with or without ICs, extended beyond C4 and encompassed other areas of periventricular intermediate zone white matter, especially C1 and C6.

In their study of human preterm infants with white matter injury, Verney et al. focused on periventricular crossroads and reported increased microglia cells and reactive astrocytes reminiscent of some of the alterations in our viral inoculation models [42]. In their investigation and our series of human neonates, ICs or cells with similar cytological features were not observed. Similarly, ICs were not identified in our series of brain sections from macaque neonates which had been subjected to experimental umbilical cord clamping and significant perinatal hypoxia. The apparent absence of ICs in these various models suggests that IC abundance in pigtail macaque fetuses, especially after maternal viral inoculation, may be specific to certain species and/or the experimental manipulations we conducted. It is possible that viral inoculation elicits responses in the dam and/or fetus (e.g., cytokine production) which secondarily lead to white matter glial cell responses and other stressors (e.g., global hypoxia) do not have the same effect. Alternatively, it may be that the timing in gestation and stage of neurodevelopment influences the vulnerability of the fetal brain to the pattern of white matter injury we describe. This vulnerability may only exist in the early third trimester and not in the neonatal period when experimental cord clamping was performed.

### IC-related glial cell activation and viral inoculation

Numerous ICs, microglial activation and reactive astrocytes were primarily observed in FLUAV or INT-ZIKA fetuses, 5 days or 20–24 days after inoculation respectively. With rare exceptions, control, SHORT-ZIKA, and LONG-ZIKA fetuses were IC poor. In LONG-ZIKA fetuses, when inoculation was done in the first or second trimester and preceded delivery by many weeks (39–97 days), the IC-rich phenotype was uncommon but, as reported elsewhere, myelination was structurally and transcriptionally altered [12]. Specifically, deep white matter showed markedly reduced expression of oligodendrocyte genes (e.g., *MBP*) involved in the formation and maintenance of myelin sheaths, reduce MBP immunoreactivity, and ultrastructural “decompaction” of myelin sheaths. It is unclear how this evidence of dysmyelination in LONG-ZIKA fetuses relates to ICs and associated glial changes reported here because the dysmyelination data was obtained from deep white matter in the superior parietal cortex, not the ICR, and at much longer inoculation-to-delivery intervals. Appropriately fixed samples were not available from the ICR of the LONG-ZIKA cohort to perform electron microscopy and transcriptional genomic studies of the ICR were not part of the current study. However, no differences in MBP immunolabeling or ultrastructural myelin compaction were identified in the ICR of any of the cohorts examined in the current study.

Nonetheless, differences between the cohorts in the timing of inoculation and delivery and prevalence of ICs and related glial alterations have some potentially important implications. IC-rich fetal brains were significantly more frequent in the FLUAV and INT-ZIKA groups and therefore the IC-rich phenotype is not specific to one pathogen. As SHORT-ZIKA dams were inoculated 3–7 days prior to necropsy, like the FLUAV cohort, it is possible that glial pathology has a time-dependent component unique to each pathogen and is relatively delayed after Zika virus inoculation. Moreover, rare IC-rich examples in the other groups, including a control fetus, indicate that these glial changes can arise in pregnancies with no known pathogen exposure. Gestational age may also be an important factor, as with one exception, all the IC-rich fetuses were less than an age of 150 gestational days. Collectively, the findings suggest that ICs are part of a non-specific pattern of glial cell activation, the etiology of which is likely diverse and may be clinically occult. The clinical consequences of the IC-rich phenotype are unclear. ICs and related glial cell changes may represent a transient, possibly protective, phenomenon, or possibly an early stage in the evolution of dysmyelination reported in LONG-ZIKA fetuses [12]. 

A growing body of evidence suggests that a variety of maternal factors, including viral infection, can affect the postnatal neurodevelopmental outcomes of offspring without dramatic gross or microscopic brain pathology [43–45]. Although conflicting data exist, multiple human epidemiological investigations have reported higher rates of schizophrenia, bipolar disorder, autism and other neurobehavioral disorders in children or adults whose mothers had influenza A infection during pregnancy (reviewed by Fung et al. [46]). Influenza A infection of pregnant mice alters postnatal expression of myelination genes and white matter fractional anisotropy in their pups, despite no evidence of fetal infection [47–49]. The congenital Zika virus spectrum encompasses a wide range of neurological outcomes, most notably severely affected fetuses with microcephaly and profound brain dysmorphology. However, more mildly affected normocephalic children are suspected [50–53], including individuals with delayed myelination [54]. In rhesus macaques, prenatal maternal Zika virus infection altered the social behavior of their offspring [55] and postnatal inoculation of 4-month-old rhesus macaques led to behavioral and cognitive changes and relatively subtle structural alterations, which were present at 12 months of age [56]. Neurodevelopmental delay was also evident in an immunocompetent rodent model of maternal Zika virus, even though virus was not detected in the brain tissue 24 h after birth (2 days post inoculation).

In our study and many of those cited in the preceding paragraph, virus was rarely detected by RT-qPCR in tissues from the fetuses; no inflammation was evident in the central nervous system or other fetal organ. Collectively, these data make it very unlikely that there is a direct relationship between the white matter alterations we observed and fetal brain viral infection. Alternatively, maternal immune activation can occur during a viral or bacterial infection in pregnancy resulting in fetal exposure to maternal inflammatory mediators [45, 57–59]. These include cytokine responses to ZIKV [60, 61], FLUAV [62], and other infectious agents [44]. We suspect that the IC-rich phenotype represents a response to cytokines or other humoral mediators which are triggered by maternal viral inoculation or other forms of maternal stress. Maternal interleukin-6 (IL-6) and other cytokines are elevated in association with Zika, Influenza A, and other infections [44, 62, 63] and have been linked to fetal white matter injury in some studies [4]. For example, maternal serum IL-6 levels during pregnancy correlate inversely with postnatal fractional anisotropy (“suggestive of reduced integrity under high inflammatory conditions”) in the uncinate fasciculus [64], a major white matter tract which courses through the IC-rich C4 crossroad region [65]. In the same study, the offspring of mothers with elevated maternal IL-6 levels had poorer measures of cognition at 12 months of age. This presumed pathogenic model could explain the infrequency of the IC-rich phenotype in controls and some of the ZIKV groups and will serve as the basis for future efforts to identify changes in correlative biomarkers.

### Strengths and limitations

The strength of this investigation is in its novel focus on the identification and description of a previously unreported neuropathological phenomenon across two different models of maternal-placental-fetal inflammation in nonhuman primates. Additionally, these models, particularly amongst the ZIKV subgroups, allowed for the association of IC development with the time interval between viral inoculation and delivery. An additional strength is the use of the pregnant NHP model to analyze the impact of maternal viral infection on fetal neurodevelopment. Not only does the pregnant NHP recapitulate many features of human pregnancy, but the timeline of fetal neurodevelopment is more similar to that in humans than for other mammalian models. One study limitation common to the case series study design is the inability to calculate incidence as the sample size is modest. Related to this limitation is a lack of data to directly link IC-related glial changes to defects in myelination and/or neurocognitive function, which exist in the LONG-ZIKA cohort and might become evident postnatally.

## Conclusions

Epidemiologic studies and animal experiments indicate that fetal exposure to a maternal infection increases the risk for neuropsychiatric disorders, but identification of subtle and common signs of fetal brain injury after a maternal infection has been elusive. Our finding of a transient cellular phenotype of astrocytes with large cellular inclusions in the deep fetal white matter after maternal viral infection is in an area of the fetal brain that is particularly susceptible to injury and exhibits high myelination and axonal growth. ICs are a hallmark of a larger spectrum of lysosomal activation within astrocytes and microglial activation. Future studies would be useful to determine if ICs or related changes within the deep fetal white matter occur in humans or other NHP models of infection in pregnancy.

## Electronic supplementary material

Below is the link to the electronic supplementary material.

Supplementary Material 1

## Data Availability

Data is provided within the manuscript or supplementary information files, additional data is available from the corresponding author upon reasonable request.

## Abbreviations

CALB2: calretinin
CASP3: caspase 3
cDMEM: complete Dulbecco’s modified Eagle’s medium
Ct: cycle threshold
CTSS: cathepsin S
DAPI: 4’,6-diamidino-2-2phenylinverdole
DIA: digital image analysis
FBS: fetal bovine serum
FLUAV: influenza A virus
GFAP: glial fibrillary acidic protein
IACUC: Institutional Animal Care Use Committee
IBA1: ionized calcium binding adapter molecule 1
ICR: inclusion cell region
ICs: inclusion cells
Ki67/MIB1: marker of proliferation Kiel67
LAMP1: lysosomal-associated membrane protein 1
LAMP2: lysosomal-associated membrane protein 2
LC3: microtubule-associated protein 1 A/1B-light chain 3, SOX2, SRY-Box 2
MBP: myelin basic protein (MBP)
NBF: neutral buffered formalin
NeuN: neuron-specific nuclear protein
NHP: nonhuman primate
NS1: ZIKV non-structural protein 1
NUP62: nucleoporin 62
OCT: optimal cutting temperature
OLIG2: oligodendrocyte transcription factor 2
PASd: periodic acid-Schiff-diastase stain
RT-qPCR: real-time quantitative polymerase chain reaction
SOX 10: SRY-Box transcription factor 10
VIS: Visiopharm Integrator System
WaNPRC: Washington National Primate Research Center
ZIKV: Zika virus
