# Supplementary material for: Granular cytoplasmic inclusions in astrocytes and microglial activation in the fetal brain of pigtail macaques in response to maternal viral infection
Source: Acta Neuropathol Commun. 2025 Mar 11;13:55. doi: 10.1186/s40478-025-01970-9 (PMC11895267; doi:10.1186/s40478-025-01970-9)
Supplement: Supplementary file 1 — Supplementary Material 1 [file 40478_2025_1970_MOESM1_ESM.pdf]

SUPPLEMENTAL INFORMATION

Table S1: Animal Information and Maximal Inclusion Cell Count 2

Table S2: Absence of Inclusion Cells in Macaque Model of Perinatal Hypoxic-Ischemic Encephalopathy 4

Table S3: Presence or Absence of Inclusion Cells in a Human Neonatal Autopsy Series 6

Table S4: Animal Endemic Pathogens by Fetal Brain Inclusion Cell Group 17

Table S5: PCR Primers and Probes 18

Table S6: Antibodies 19

Table S7: Detection of ZIKV or IAV (Flu) Viral RNA or Infectious Virus by Fetal Brain Inclusion Cell Group in Fetal and Placental Tissues 20

Figure S1: Graphic Illustration of Study Design 22

Figure S2: GFAP Immunolabeling in Inclusion Cell-Rich Region 23

Figure S3: Comparative Autofluorescence of Lipofuscin and Lipofuscin-like Inclusion Granules 24

Figure S4: Gestational Age at Time of Delivery versus Maximal IC Count 25

Figure S5: Representative Images of LAMP2 Immunohistochemistry 26

Figure S6: Focal Inclusion Cell Aggregates in Thalamus 27

Figure S7: Electron Micrographs of Neuropil with Early Myelination 28

Figure S8: Zika Viral Protein NS1 Immunohistochemistry 29

Table S1: Animal Information and Maximal Inclusion Cell Count

Group	ID	Pathogen	Inoculation-Delivery Interval (Days)	Maternal Age at Necropsy (Years)	Gestational Age at Inoculation (Days)	Fetal Age at Delivery (Days)	Fetal Sex	Fetal Weight	Fetal Brain Weight	Maximal IC Count
LONG-ZIKA	ZIKA1	ZIKV*	43	9	116	159	M	453	NR	0
	ZIKA2	ZIKV*	77	5	83	159	F	451.1	NR	0
	ZIKA3	ZIKV**	87	10	63	150	F	385	51.4	0
	ZIKA4	ZIKV**	82	12	60	142	F	451	NR	11
	ZIKA5	ZIKV**	97	9	60	157	F	452.8	59.4	0
INT-ZIKA	ZIKA8	ZIKV**	20	7	121	141	F	316.6	47.2	5
	ZIKA10	ZIKV**	22	13	138	160	F	563.3	56.3	0
	ZIKA11	ZIKV**	21	6	125	146	F	479.8	53.3	78
	ZIKA12	ZIKV**	21	11	114	135	M	388.6	50.6	95
	ZIKA13	ZIKV**	21	13	115	136	F	351.6	42.8	65
	ZIKA14	ZIKV**	21	15	124	145	F	404.4	58.5	6
	ZIKA19	ZIKV**	24	7	115	139	F	350.8	52.5	3
SHORT-ZIKA	ZIKA9	ZIKV**	7	14	138	145	F	381.7	42.6	7
	ZIKA15	ZIKV**	3	10	146	149	M	404.7	58.1	12
	ZIKA16	ZIKV**	3	12	149	152	M	426.5	NR	0
	ZIKA17	ZIKV**	2	5	150	152	M	372.4	52.1	0
	ZIKA18	ZIKV**	4	9	153	157	M	362.5	55.9	0
FLUAV	FLU1	FLUAV H1N1^	5	5	125	130	M	291	43.8	94
	FLU2	FLUAV H1N1^	5	12	126	131	F	297.2	41.1	82
	FLU6	FLUAV H1N1^	5	8	124	129	F	267.9	45.6	12
	FLU7	FLUAV H1N1^	5	5	126	131	F	326	42.1	3
	FLU8	FLUAV H1N1^	5	5	127	132	F	258.3	42.1	8
	FLU9	FLUAV H1N1^	5	13	138	143	F	358	45.8	0
	FLU12	FLUAV H3N2^^	5	4	126	131	F	316.9	42.5	58
	FLU13	FLUAV H1N1^	5	9	134	139	F	372.1	49.7	12

Group	ID	Pathogen	Inoculation-Delivery Interval (Days)	Maternal Age at Necropsy (Years)	Gestational Age at Inoculation (Days)	Fetal Age at Delivery (Days)	Fetal Sex	Fetal Weight	Fetal Brain Weight	Maximal IC Count
	FLU14	FLUAV H1N1^	5	6	135	140	M	334.2	50.1	32
	FLU17	FLUAV H1N1^	5	7	127	132	M	277.1	40.7	7
Controls	CTRL1	None	100	13	59	159	F	431.9	57.8	0
	CTRL2	None	92	11	62	156	F	467.2	56.3	0
	CTRL3	None	58	7	99	157	F	351	NR	0
	CTRL5	None	No inoculation	7	No inoculation	156	F	NR	NR	1
	CTRL13	None	20	5	138	158	F	499.2	55.9	0
	CTRL14	None	23	9	132	155	M	573	60.1	0
	CTRL15	None	21	9	133	154	F	411.9	62.1	8
	CTRL24	None	20	9	127	147	M	469	61.3	4
	CTRL25	None	3	11	141	144	M	412.6	57.7	4

Abbreviations: IC, inclusion cell; NR, not recorded

*Zika virus Cambodia (FSS13025), 2010; GenBank Accession Number: KU955593

**Zika virus Brazil (Fortaleza), 2015; GenBank Accession Number: KX811222.1

^Influenza A virus (A/California/07/2009 (H1N1)); GenBank Accession Numbers: CY121682 (hemagglutinin) and CY266193 (neuraminidase)

^^Influenza A virus (A/Texas/71/2017(H3N2); GenBank Accession Numbers: CY250324.1 (hemagglutinin gene) and CY242128.1 (neuraminidase gene)

Table S2: Absence of Inclusion Cells in a Macaque Model of Perinatal Hypoxic-Ischemic Encephalopathy[#]

ID	Age	Level*	Brain Pathology	Inclusion Cells**
1	6 mo	-4 mm		0
6	6 mo	-3 mm		0
8	6 mo	-3 mm		0
10	6 mo	-3 mm		0
12	6 mo	-6 mm		0
15	6 mo	-10 mm		0
26	6 mo	-7 mm		0
22	Day 3	-13 mm	Devastating cortical and deep gray matter necrosis with severe gliosis	0
28	Day 3	-6 mm	Infarct in ventral lateral thalamus	0
		-10 mm	Infarct in rostral brainstem	0
17	Day 4	-9 mm	Ventral lateral thalamic infarct with gliosis	0 (PASd)
		-10 mm	Ventral lateral thalamic infarct with gliosis	0 (PASd)
		-10 mm		0
		-13 mm		0
3	6 mo	-6 mm		0
7	6 mo	-6 mm		0
11	6 mo	-4 mm		0
13	6 mo	-5 mm		0
18	6 mo	-7 mm		0
19	6 mo	-5 mm		0
21	6 mo	-5 mm		0
2	Day 3	-4 mm		0
20	Day 3	-8 mm	Ventral lateral thalamic infarct with gliosis	0 (PASd)
5	Day 4	-3 mm		0
		-7 mm		0
		-12 mm		0
9	Day 7	-8 mm	Meningitis	0
		-6 mm	Meningitis	0
		-12 mm	Meningitis	0
4	6 mo	-6 mm		0
14	6 mo	-3 mm		0
23	6 mo	-7 mm		0
24	6 mo	-5 mm		0
25	6 mo	-5 mm		0
27	6 mo	-5 mm		0
29	6 mo	-6 mm		0

Abbreviations: Epo, erythropoietin; UCO, umbilical cord occlusion; PASd, periodic acid-Schiff-diastase; TH, therapeutic hypothermia

*Coronal level based on the macaque brain atlas: Martin RF, Bowden DM (2000) Primate Brain Maps: Structure of the Macaque Brain, Amsterdam: Elsevier Science; http://braininfo.rprc.washington.edu/primatebrainmaps/atlas/Mapindex.html).

**Inclusion cells excluded by examination of hematoxylin-and-eosin sections, along with diastase pretreated periodic acid-Schiff sections as indicated.

#Pigtail macaques *(Macaca nemestrina)* were delivered 1-8 days prior to term (168 +/- 2 days) immediately after 18-20 minutes UCO with or without cord clamping (control). Some animals received no treatment, and others were treated with TH +/- intravascular Epo. For details see:

Wood TR, Vu PT, Comstock BA, Law JB, Mayock DE, Heagerty PJ, Burbacher T, Bammler TK, Juul SE. Cytokine and chemokine responses to injury and treatment in a nonhuman primate model of hypoxic-ischemic encephalopathy treated with hypothermia and erythropoietin. *J Cereb Blood Flow Metab*. 2021 Aug;41(8):2054-2066. doi: 10.1177/0271678X21991439. Epub 2021 Feb 7. PMID: 33554708; PMCID: PMC8327104.

Table S3: Presence or Absence of Inclusion Cells in a Human Neonatal Autopsy Series

ID	Gestational Age at Birth (wks)	Age at Death	Sex	Post-mortem interval (hrs)	Malformation / Anomaly		Other Diagnoses		Sampling of IC-Rich Region*	Sampling of Other Areas**	Inclusion Cells Present?
					CNS	Non-CNS	CNS	Non-CNS			
1	40	9 d	F	31	HIE, brain injury		Ischemia-infarction	Liver steatosis	+	+	No
2	42	6 h	F	78	HIE	Cardiac respiratory failure, coagulopathy, anemia, severe metabolic acidosis		In-utero feto-maternal hemorrhage	+	+	No
3	34	18 d	M	4.75	Smith-Lemli-Opitz syndrome		Deep white matter infarct	Splenomegaly, anasarca	+	+	No
4	33	7 w	F	14	Epilepsy (focal apoptotic neurons within DG)	Severe pulmonary hypertension, veno-occlusive disease	Seizure		+	+	No
5	24	13 d	M	15		Idiopathic intestinal perforation with clinical necrotizing enterocolitis	Intraventricular hemorrhage, PVL	Myocardial infarct, hepatic cholestasis	+	+	No
6	34	19 d	F	28	Frontal lobe fore shortening, mild hydrocephalus	Necrotizing enterocolitis, complete atrioventricular canal defect, Trisomy21			+	+	No
7	27	7 w	M	12.5	Hypoxic ischemic injury with PVL			Multi-organ hypoxic ischemic injury	+	+	No

ID	Gestational Age at Birth (wks)	Age at Death	Sex	Post-mortem interval (hrs)	Malformation / Anomaly		Other Diagnoses		Sampling of IC-Rich Region*	Sampling of Other Areas**	Inclusion Cells Present?
					CNS	Non-CNS	CNS	Non-CNS			
8	32	2 m	F	58		Cardiopulmonary abnormalities, congenital cardiac anomalies	PVL with acute hypoxic ischemic injury	Renomegaly	+	+	No
9	39	3 w	F	16.5		Musculoskeletal abnormalities, palpable fractures	Small subdural hemorrhage occipitally, mild gliosis		+	+	No
10	40	22 d	M	75		Pterygium syndrome and acute respiratory failure, arthrogryposis multiplex	Bilateral remote parenchymal injury, generalized mild gliosis		+	+	No
11	40	4 w	F	19		HLH and supracardiac total anomalous pulmonary venous return, congenital heart disease		Coagulopathy, multi-organ failure (microinfarcts in hippocampus and other areas)	+	+	No
12	32	7 d	F	15	Subdural hematoma, diffuse hypoxic-ischemic injury with widespread gliosis and early mineralization	Cystic necrosis of liver, respiratory failure	PVL, intraventricular hemorrhage, few pyknotic and karyorrhectic cells are noted in hippocampus	Congested spleen	+	+	No
13	40	1 w	M	45	Acute chronic HIE, PVL with prominent gliosis and neuronal loss			Congestion and hemorrhage	+	+	No

ID	Gestational Age at Birth (wks)	Age at Death	Sex	Post-mortem interval (hrs)	Malformation / Anomaly		Other Diagnoses		Sampling of IC-Rich Region*	Sampling of Other Areas**	Inclusion Cells Present?
					CNS	Non-CNS	CNS	Non-CNS			
14	33	8 w	M	10.5	Small brain (36 gm) with mild white matter atrophy and gliosis, PVL	Heterotaxy syndrome, complex congenital heart disease			+	+	No
15	38	3 w	F	57.5		Congenital diaphragmatic hernia, coagulopathy	Diffuse edema and gliosis	Liver, spleen and kidney defects	+	+	No
16	34	18 d	M	13.5		Beckmann-Wiedemann Syndrome, respiratory failure, acute kidney injury	Kernicterus involving hippocampi, diffuse gliosis with PVL	Thymic cortical stress	+	+	No
17	35	3 w	F	12.5	Mild ventriculomegaly	Anasarca, cardiovascular anomalies	Parietooccipal cerebral infarct with subarachnoid hemorrhage, diffuse gliosis		+	+	No
18	39	3 w	M	2.75		Alveolar capillary dysplasia with pulmonary vein misalignment, complex congenital heart disease			+	+	No

ID	Gestational Age at Birth (wks)	Age at Death	Sex	Post-mortem interval (hrs)	Malformation / Anomaly		Other Diagnoses		Sampling of IC-Rich Region*	Sampling of Other Areas**	Inclusion Cells Present?
					CNS	Non-CNS	CNS	Non-CNS			
19	28	15 d	F	6		CHD		Acute multifocal pneumonia, congestion and hemorrhage	+	+	No
20	28	5 w	F	17.5		NEC	Dural vein thrombosis, hemorrhagic cerebral and cerebellar necrosis, germinal matrix hemorrhage, multiple seizures		+	+	No
21	28	3 w	M	38.5		NEC, RDS, presumed sepsis		CLD, multiple organ defects	+	+	No
22	41	2 d	F	21.5	Severe hypoxia	Acute chorioamnionitis	Cerebral edema, possible seizure		-	+	No
23	38	2 d	F	7	Severe hypoxia	Multiple congenital anomalies		Bilateral pneumo-thoraces	-	+	No
24	33	3 d	M	40		Congenital "solitary" hepatic hemangioma, cardiomegaly		Lung vascular congestion, multiple foci of acute hemorrhage and necrosis consistent with coagulopathy	-	+	No
25	38	4 d	M	38	Hypoxic-ischemic injury	Severe acidosis, cardiorespiratory failure	Seizure	Visceral anomalies	-	+	No

ID	Gestational Age at Birth (wks)	Age at Death	Sex	Post-mortem interval (hrs)	Malformation / Anomaly		Other Diagnoses		Sampling of IC-Rich Region*	Sampling of Other Areas**	Inclusion Cells Present?
					CNS	Non-CNS	CNS	Non-CNS			
26	19	0	M	13.25		Nondysmorphic fetus with amniotic fluid infection			-	+	No
27	28	14 d	M	16	Mild changes consistent with hypoxic-ischemic CNS injury	Massive subacute hepatic necrosis with iron overload, coagulopathy	Mild, diffuse gliosis in white matter		-	+	No
28	unknown	3 y	M	2	Anoxic brain injury secondary to pulmonary arrest, HIE		Cerebral edema, status epilepticus		-	+	No
29	37	2 m	F	8		Complex congenital heart disease, cardiomegaly	Diffuse mild cerebral white matter gliosis	Aspiration pneumonitis	-	+	No
30	37	3 y	M	20.5	Global developmental delay, hypoxic-ischemic injury with edema	Myopathy, cardiac failure, respiratory failure		Infectious diseases, respiratory distress, sepsis	-	+	No
31	35	4 w	M	15.5	Severe HIE	Multi-organ failure		Neonatal herpes infection, increased iron deposition in viscera	-	+	No
32	36	2 d	F	48		Autosomal recessive bilateral polycystic kidney disease and respiratory failure		Adrenal hemorrhage	-	+	No

ID	Gestational Age at Birth (wks)	Age at Death	Sex	Post-mortem interval (hrs)	Malformation / Anomaly		Other Diagnoses		Sampling of IC-Rich Region*	Sampling of Other Areas**	Inclusion Cells Present?
					CNS	Non-CNS	CNS	Non-CNS			
33	28	8 w	F	16.5	Remote and acute HIE	Chronic lung disease		Multiple congenital anomalies	-	+	No
34	38	3	M	15.75		Hemorrhagic and necrotic small bowel, anomalies in alimentary tract, liver failure	Mild edema and hypoxic injury		-	+	No
35	29	6 w	M	14	Global hypoxic/ischemic CNS injury, intraventricular hemorrhage and diffuse PVL	Pulmonary and cardiac abnormalities			-	+	No
36	33	5 w	F	84		NEC			-	+	No
37	36	10 d	F	23		HLH, coagulopathy	Large ICH, disrupted leptomeninges	Multicystic dysplasia	-	+	No
38	"term"	18 h	M	14		Complex CHD, total anomalous pulmonary venous return		Lymphatic distention	-	+	No
39	25	7 w	M	14	Small brain, severe global hypoxic ischemic injury, PHH	NEC, intra-gastric organizing hematoma			-	+	No
40	31	4 w	M	32		Rh-isoimmunization hydrops fetalis, liver failure		Respiratory distress	-	+	No
41	32	10 w	M	11		Liver dysfunction of uncertain etiology	CMV infection		-	+	No

ID	Gestational Age at Birth (wks)	Age at Death	Sex	Post-mortem interval (hrs)	Malformation / Anomaly		Other Diagnoses		Sampling of IC-Rich Region*	Sampling of Other Areas**	Inclusion Cells Present?
					CNS	Non-CNS	CNS	Non-CNS			
42	37	4 w	F	24		Truncus arteriosus	Mild HIE with mild gliosis		-	+	No
43	40	20 d	M	11	Acute HIE, SAH	Severe refractory pulmonary hypertension, hemolysis, coagulopathy			-	+	No
44	27	4 d	F	6 days	Shock/ischemia, widespread encephalopathy			Splenic congestion	-	+	No
45	40	11 d	F	58	ICH secondary to a vein of Galen malformation		Ischemic brain injury, hydrocephalus		-	+	No
46	35	10 d	M	14.5	Hypoxic-ischemic brain injury	Bilateral multicystic dysplastic kidneys, NEC	Neuronal loss in brainstem, pons, hippocampus, gliosis		-	+	No
47	41	9 d	F	4 days	HIE, episodic multi-focal seizure		Diffuse edema, seizure		-	+	No
48	34	6 d	F	20	Encephalopathy with hypertonia, loss of brainstem reflexes	Prematurity, mild lung edema			-	+	No
49	42	31 h	F	31		Bacteremia	Brain with mild microglial activation with a small focus of PVL		-	+	No
50	35	8 d	M	66	CNS hypoxic/ischemic injury, white matter gliosis, PVL	Severe multiorgan dysfunction			-	+	No

ID	Gestational Age at Birth (wks)	Age at Death	Sex	Post-mortem interval (hrs)	Malformation / Anomaly		Other Diagnoses		Sampling of IC-Rich Region*	Sampling of Other Areas**	Inclusion Cells Present?
					CNS	Non-CNS	CNS	Non-CNS			
51	40	3 d	F	40.5		Profound hypoxemic respiratory failure, lung developmental arrest			-	+	No
52	35	3 w	M	19	Acute hypoxic/ischemic brain injury	Congenital heart disease		Medullary hemorrhage	-	+	No
53	39	16 d	F	70	Hypoxic/ischemic injury, diffuse WM gliosis	Congenital heart disease		Clinical hypoxia, multi-organ failure	-	+	No
54	37	1 d	F	14	Large occipital encephalocele, focal dysplasia	Multiple congenital abnormalities	Poorly formed hippocampi, neuronal disorganization, hypoxic-ischemic injury		-	+	No
55	34	22 d	M	8.5		Congenital liver tumor, hypothyroidism		Intra-abdominal hemorrhage, ischemic necrosis	-	+	No
56	38	20 d	F	81.5	Encephalopathy	Arthrogryposis multiplex congenita, pulmonary hypoplasia	Irregular gliosis and increase in microglia (GFAP, CD68)	Respiratory distress	-	+	No
57	40	4 d	F	15	Severe global HIE			Patchy adrenal hemorrhage and infarction	-	+	No
58	36	16 d	M	10	Hypoxic/ischemic injury, diffuse WM gliosis, PVL	Complex CHD, status post-surgical repair	SAH		-	+	No
59	33	13 d	M	63		Congenital alveolar dysplasia		Interstitial chromosomal deletion ch17	-	+	No

ID	Gestational Age at Birth (wks)	Age at Death	Sex	Post-mortem interval (hrs)	Malformation / Anomaly		Other Diagnoses		Sampling of IC-Rich Region*	Sampling of Other Areas**	Inclusion Cells Present?
					CNS	Non-CNS	CNS	Non-CNS			
60	40	11 d	M	47		Meconium aspiration syndrome, total anomalous pulmonary venous return		Pulmonary hemorrhage	-	+	No
61	32	17 d	F	78.5	Remote hypoxic-ischemic brain damage, gliosis, IVH, encephalopathy	Bowel obstruction, renal failure	PVL	Edema, jaundice, focal pneumonia, multi-organ failure	-	+	No
62	36	2 d	M	36		*Escherichia coli* sepsis	IVH and intraparenchymal hemorrhage, edema	Respiratory distress	-	+	No
63	38	3 w	F	15	Hypoxic-ischemic brain injury	Cardiac respiratory failure, Lactic acidemia, enlarged heart		Generalized edema	-	+	No
64	35	6 d	M	69	Subicular necrosis, acute HIE	22q11.2 chromosomal deletion, Di George syndrome			-	+	No
65	35	6 w	F	21		Congenital cardiomyopathy		Multi-organ failure	-	+	No
66	39	3 d	F	5 days		Fetal distress, prenatal hydrops	ICH	Coagulopathy	-	+	No
67	35	4 w	F	21	Remote HIE	CLD		Multi-organ congestion and hemorrhage	-	+	No
68	37	8 d	M	42	Brain herniation	Ornithine transcarbamylase deficiency, hyperammonemia	Diffuse cerebral edema, seizures	HSM	-	+	No

ID	Gestational Age at Birth (wks)	Age at Death	Sex	Post-mortem interval (hrs)	Malformation / Anomaly		Other Diagnoses		Sampling of IC-Rich Region*	Sampling of Other Areas**	Inclusion Cells Present?
					CNS	Non-CNS	CNS	Non-CNS			
69	37	7 d	M	31		*Clostridium perfringens* sepsis, bowel perforation and hemolysis, Charge syndrome			-	+	No
70	25	23 d	M	16.25		NEC	Severe ICH	Severe pneumonia, pulmonary hemorrhage	-	+	No
71	26	3 w	M	23		NEC and pneumatosis	IVH, PVL	Sepsis	-	+	No
72	36	5 d	F	58	HIE (pontosubicular necrosis, white matter gliosis)	Caudal regression syndrome, duodenal atresia		Pulmonary hypoplasia	-	+	No
73	40	3 w	M	49	HIE (pontosubicular necrosis) and *Candida albicans*	Neonatal HSV, HLH, and disseminated *Candida albicans*	Two small infarcts	Massive liver necrosis; disseminated fungi	-	+	No
74	36	17 h	M	50		Bilateral renal agenesis	Acute cerebellar hemorrhage	Infantile respiratory distress with hyaline membrane disease	-	+	No
75	39	4 w	F	15	CNS injury	Cardiac anomalies, arrest		Renal medullary injury	-	+	No
76	31	10 d	M	43		NEC		Candida infection, mild SAH and occipital hemorrhage	-	+	No

ID	Gestational Age at Birth (wks)	Age at Death	Sex	Post-mortem interval (hrs)	Malformation / Anomaly		Other Diagnoses		Sampling of IC-Rich Region*	Sampling of Other Areas**	Inclusion Cells Present?
					CNS	Non-CNS	CNS	Non-CNS			
77	34	2 w	F	63	Global hypoxic/ ischemic injury	Coagulopathy, CLD, complex CHD	Massive CNS deep white matter infarction	HSM	-	+	No
78	36	3 d	M	2.5 days	ICH, hydrocephalus	Liver failure, severe coagulopathy, metabolic acidosis	Massive CNS hemorrhage and infarction		-	+	No

Abbreviations: CHD, congenital heart disease; CLD, chronic lung disease; CMV, cytomegalovirus; CNS, central nervous system; HIE, hypoxic ischemic encephalopathy; HLH, hypoplastic left heart; HSM, hepatosplenomegaly; HSV, herpes simplex virus; ICH, intracranial hemorrhage; IVH, intraventricular hemorrhage; NEC, necrotizing enterocolitis; PHH, post- hemorrhagic hydrocephalus; PVL, periventricular leukomalacia; SAH, subarachnoid hemorrhage; RDS, respiratory distress syndrome

* Comparable site to Inclusion Cell-Rich area in non-human primates in the deep white matter adjacent to the lateral geniculate nucleus.

** Sampling of other areas of the deep white matter.

Table S4: Animal Endemic Pathogens by Fetal Brain Inclusion Cell Group

Test Result	IC Density	Chagas	CHIKV	DENV	Herpes B-virus	SFV	SIV	SRV-1	SRV-2	STLV-1	Valley Fever
Neg	IC-Poor	9	1	7	14	10	19	2	16	19	11
	IC-Rich	11	3	7	12	10	17	0	16	17	8
Pos	IC-Poor	4	1	1	5	1	0	1	3	0	2
	IC-Rich	6	0	0	5	1	0	0	1	0	2
Not tested	IC-Poor	6	17	11	0	8	0	16	0	0	6
	IC-Rich	0	14	10	0	6	0	17	0	0	7
p-value*	-	1	0.400	1	1	1	1	1	0.605	1	1

Abbreviations: IC, inclusion cell; Chagas, *Trypanosoma cruzi*; CHIKV, Chikungunya virus; DENV, dengue virus; Herpes B-virus (*Macacine alphaherpesvirus 1*; McHV-1; formerly *Macacine herpesvirus 1*, *Cercopithecine herpesvirus 1*, CHV-1); SFV, simian foamy virus; SIV, simian immunodeficiency virus; SRV-1, simian retrovirus 1; SRV-2, simian retrovirus 2; STLV-1, simian T-Cell leukemia virus 1; Valley fever, coccidiomycoides.

*No significant difference was identified in the proportion of a maternal positive versus negative endemic pathogen between IC-poor and IC-rich groups (Fisher exact test). Note that animals that were not tested for a particular pathogen were not included in this comparison.

Table S5: PCR Primers and Probes

Primer/ Probe Name	Pathogen	Direction	Sequences	Size(bp)	Amplicon Length (bp)	Target gene, position
Caspid 162 FWD	ZIKV	F	5’-GAGATTCACGGCAATCAAG-3'	19	144	Capsid, 269-287
Caspid 287 REV	ZIKV	R	5’-CTTCTTCTCCTTCCTAGCAT -3’	20	144	Capsid, 413--394
Caspid 2042 Probe	ZIKV	R	5'-[56-FAM] ATTCTCAGCATGGCAGCCAGATCT [3BHQ_1]-3'	24	144	Capsid, 388-365
HKUqSW Fwd Human	FLUAV H1N1	F	5’-GGGTAGCCCCATTGCAT-3'	17	177	Segment 4-HA, 188-204
HKUqSW Rev Human	FLUAV H1N1	R	5’-AGAGTGATTCACACTCTGGATTTC -3’	24	177	Segment 4-HA, 262-239
HKUqSW Probe Human	FLUAV H1N1	F	5'-[56-FAM] TGGGTAAATGTAACATTGCTGGCTGG [36-TAMsp]-3'	23	177	Segment 4-HA, 206-231

Abbreviations: HA, hemagglutinin; F, forward primer; FLUAV, influenza A virus; R, reverse primer; ZIKV, Zika virus

Table S6: Antibodies

Antibody	Source (catalog#)	Species	Dilution	Immunostainer Settings			Target
				Condi-tioning (min)	Primary Incubation (min)	Temp (°C)	
LAMP1	OriGene (TA811973)	Mouse monoclonal	1:100	32	60	36	Lysosomes
LAMP2	Santa Cruz Biotechnology (sc-18822)	Mouse monoclonal	1:100	8	16	36	Lysosomes
GFAP	Dako (MO761)	Mouse monoclonal	1:800	32	32	36	Astrocytes
IBA-1	Wako (019-19741)	Rabbit polyclonal	1:4000	32	8	36	Microglia
CTSS	Santa Cruz Biotechnology (sc-271619)	Mouse monoclonal	1:400	32	32	36	Microglia
LC3	Medical and Biological Laboratories (PM036)	Rabbit polyclonal	1:500	8	16	36	Autophagy Marker
SOX2	Invitrogen (PA1-094)	Rabbit polyclonal	1:250	32	32	37	Astrocytes, neural stem cells
SOX10	Cell Marque (3834-15)	Rabbit polyclonal	1:500	64	16	36	Oligodendro-glia
OLIG2	Immuno-biological Laboratories (18953)	Rabbit polyclonal	1:500	64	16	36	Oligodendro-glia
MBP	Millipore (MAB 382)	Mouse monoclonal	1:200	32	16	36	Myelin
Ki67 / MIB1	Dako (M7240)	Mouse monoclonal	1:100	32	16	36	Proliferative cells
NF	Dako (MO762)	Mouse monoclonal	1:400	8	16	rt	Axons
NeuN	Millipore (MAB377)	Mouse monoclonal	1:2000	32	32	36	Neurons
Calretinin	Zymed (SP65)	Rabbit polyclonal	1:2500	16	36	37	Neurons
p62	Abnova (H00008878-M01)	Mouse Monoclonal	1:1000	8	16	36	Autophagy Marker
Caspase 3	R&D Systems (MAB835)	Rabbit monoclonal	1:500	32	32	37	Apoptosis Marker
Zika virus	Arigabio (ARG65781)	Mouse Monoclonal	1:2000	32	32	36	Zika virus NS1 protein

Table S7: Detection of ZIKV or IAV (Flu) Viral RNA or Infectious Virus by Fetal Brain Inclusion Cell Group in Fetal and Placental Tissues

ID	Positive Fetal and Placental Tissues and Fluids	Negative Fetal and Placental Tissues and Fluids
IC Rich (N=17)		
FLU1	Placenta	Brain stem, cerebrum, heart, liver, lung, meninges, spinal cord, spleen, thymus
FLU2	None	Brain stem, cerebrum, heart, liver, lung, meninges, spinal cord, spleen, thymus
FLU6	Placenta	Brain stem, cerebrum, heart, liver, lung, spinal cord, spleen, thymus
FLU8	None	Brain stem, cerebrum, heart, liver, lung, placenta, spinal cord, spleen, thymus
FLU12	None	Cerebrum
FLU13	None	Cerebrum, heart, lung, placenta, spinal cord, spleen, thymus
FLU14	None	Cerebrum, heart, lung, placenta, spinal cord,spleen thymus
FLU17	Placenta	Cerebrum, lung, spinal cord, spleen, thymus
ZIKA4	None	Cerebrum, gonad, kidney, liver, placenta, spinal cord, spleen
ZIKA8	None	Brain stem, cerebrum, eye, gonad, liver, spinal cord, placenta
ZIKA9	Placenta	Brain stem, cerebrum, eye, gonad, kidney, liver, spinal cord, spleen
ZIKA11	Placenta**	Brain stem, cerebrum, eye, gonad, kidney, liver, meninges, mesenteric lymph node, spinal cord, spleen
ZIKA12	None	Brain stem, cerebrum, eye, gonad, kidney, liver, meninges, placenta, spinal cord, spleen
ZIKA13	Placenta	Brain stem, cerebrum, eye, gonad, kidney, liver, meninges, spinal cord, spleen
ZIKA14	Liver, placenta	Brain stem, cerebrum, eye, gonad, meninges, spinal cord, spleen
ZIKA15	None	Brain stem, cerebrum, eye, gonad, kidney, liver, meninges, placenta, spinal cord, spleen
CTRL15	None	None
IC Poor (N=19)		
FLU7	None	Brain stem, cerebrum, heart, liver, lung, placenta, spinal cord, spleen, thymus
FLU9	None	Brain stem, cerebrum, heart, liver, lung, placenta, spinal cord, spleen, thymus
ZIKA1	Cerebrum, meninges, spinal cord	Gonad, kidney, liver, placenta, spleen
ZIKA2	Liver, placenta	Cerebrum, gonad, kidney, meninges, spinal cord, spleen

ID	Positive Fetal and Placental Tissues and Fluids	Negative Fetal and Placental Tissues and Fluids
ZIKA3	None	Brain stem, gonad, kidney, liver, meninges, placenta, spinal cord, spleen
ZIKA5	None	Cerebrum, kidney, liver, meninges, placenta, spinal cord, spleen
ZIKA10	None	Brain stem, cerebrum, eye, gonad, kidney, liver, meninges, placenta, spinal cord, spleen
ZIKA16	Meninges, placenta	Brain stem, cerebrum, eye, gonad, kidney, liver, spinal cord, spleen
ZIKA17	Placenta**, plasma	Brain stem, cerebellum, cerebrum, eye, gonad, liver, meninges, spinal cord
ZIKA18	Placenta**	Brain stem, cerebellum, eye, gonad, liver, meninges, spinal cord
ZIKA19	None	Brain stem, cerebellum, cerebrum, eye, gonad, liver, meninges, placenta
CTRL1	None	Brain stem, cerebrum, kidney, liver, spinal cord, spleen
CTRL2	None	Brain stem, cerebrum, gonad, kidney, liver, spinal cord, spleen
CTRL3	None	Brain stem, cerebrum, gonad, kidney, liver, spleen
CTRL5	None	None
CTRL13	None	Brain stem, cerebrum, gonad, kidney, liver, placenta, spinal cord, spleen
CTRL14	None	Brain stem, cerebrum, gonad, kidney, liver, placenta, spinal cord, spleen
CTRL24	None	None
CTRL25	None	None

** Positive by plaque assay only.

Abbreviations: IC, inclusion cell

Viral load was tested by either qPCR, plaque assay, or TCID50. For Control and Zika animals, the viral qPCR target was ZIKV capsid. For FLUAV animals, the viral qPCR target was the H1N1 or H3N2 capsid. All maternal nonhuman primates infected with ZIKV had positive maternal plasma samples 2 days after inoculation except for ZIKA1 and ZIKA3. All maternal nonhuman primates infected with H1N1 had positive maternal lung samples. If any aliquot from an organ was tested positive, the whole organ was considered positive.

Figure S1: Graphic Illustration of Study Design

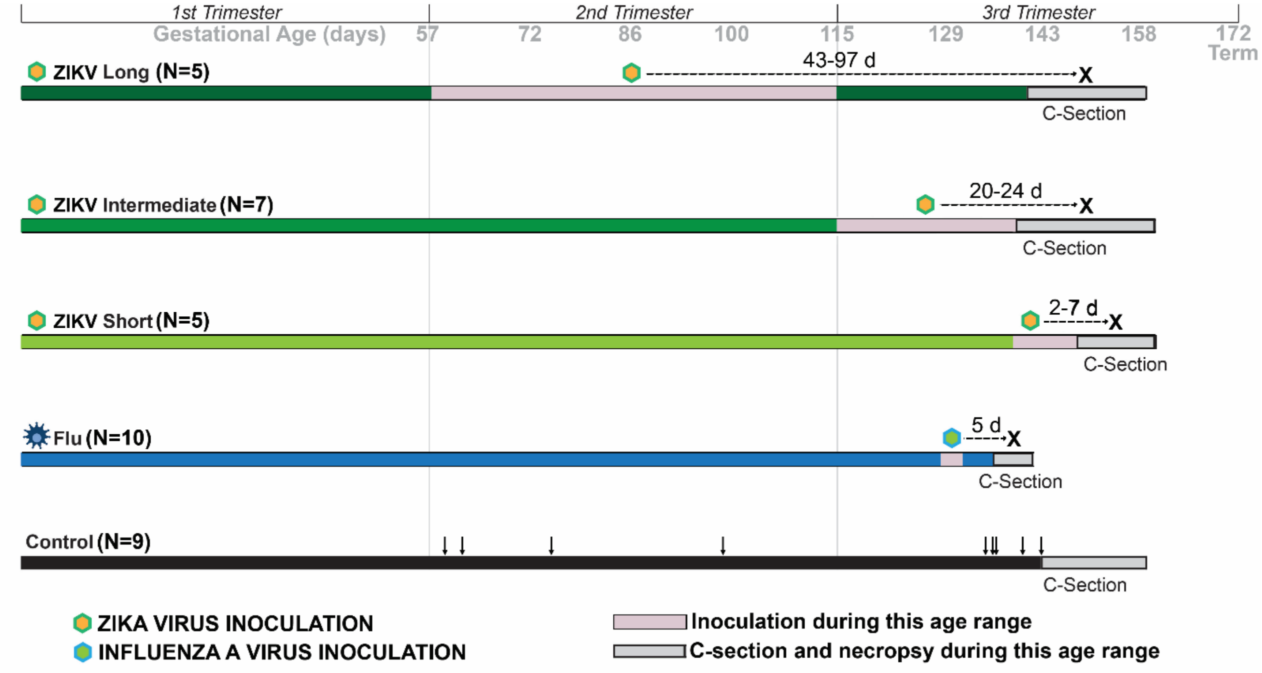

A graphic illustration of the study design demonstrates different intervals (numbers above dashed lines) between inoculation (pink bars) of pregnant dams and Cesarean section (C-section)/ fetal necropsy (gray bars) in the experimental groups. Term gestation in the pigtail macaque is 172 days. Control (sham) inoculations were performed at a range of gestational ages with each arrowhead indicating the approximate inoculation timing for a given control corresponding to those represented in the other groups. All Cesarean sections were performed in the third trimester.

Figure S2: GFAP Immunolabeling in Inclusion Cell-Rich Region

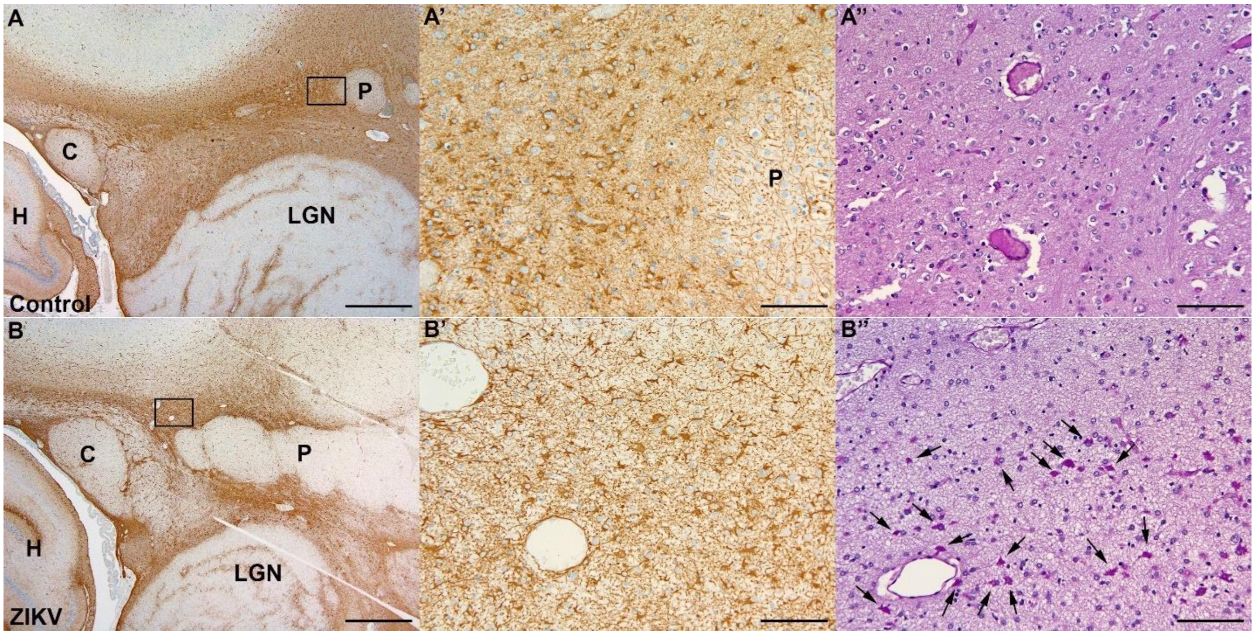

In the deep white matter location region where inclusion cells, when present, are most consistently observed and numerous, there was a similar pattern of GFAP immunoreactivity and distribution of GFAP-immunoreactive astrocytes is present in control (sham viral inoculation, A and A') and ZIKV (B and B') or FLUAV (not shown) fetuses. The rectangles in A and B indicate areas shown at higher magnification in A' and B' respectively. (A'', B") In the same regions of adjacent PASd-stained sections, abundant inclusion cells (arrows in B'') are present in the virus-exposed fetus, but not the control. Abbreviations: P, putamen, C, caudate nucleus, H, Hippocampus, LGN, lateral geniculate nucleus. Scale bars: A,B: 1 mm; A', A'', B', B'': 200 μm.

Figure S3: Comparative Autofluorescence of Lipofuscin and Lipofuscin-like Inclusion Granules

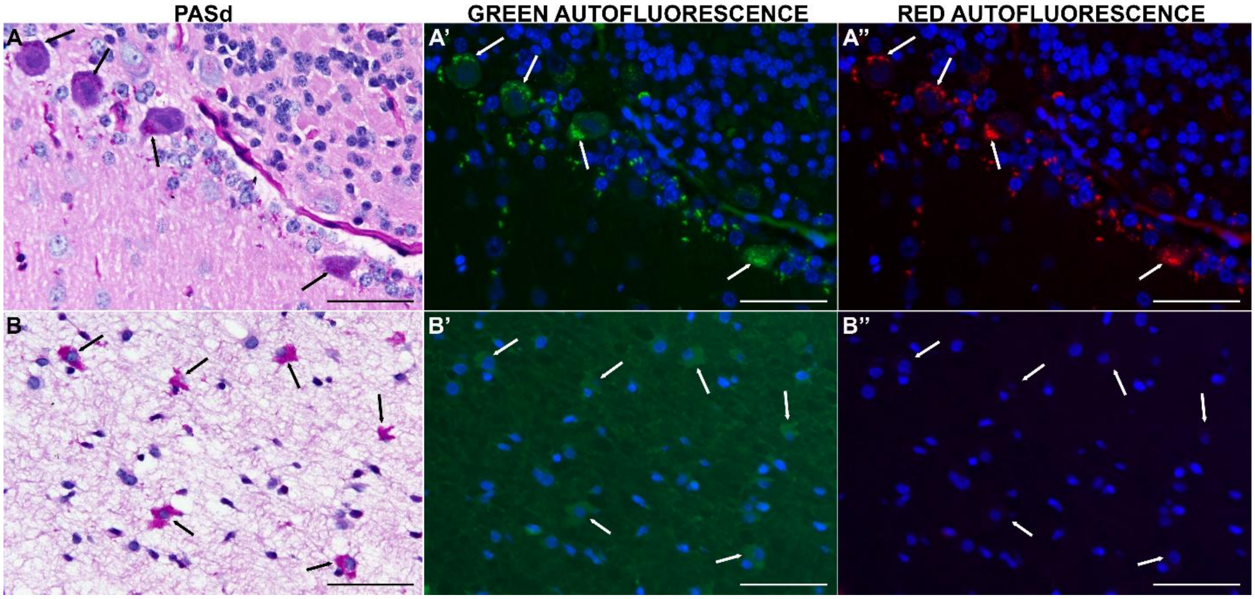

This figure illustrates the comparative autofluorescence of lipofuscin and lipofuscin-like inclusion granules from the cerebellum of a dam in the control cohort (A) and FLUAV cohort (B). (A) PASd-positive lipofuscin granules in Purkinje cells (arrows) in the cerebellum of an adult *M. nemestrina* autofluorescence bright green (A') or red (A'') with appropriate excitation and emission filters. (B) By contrast, the lipofuscin-like granules found in astrocytes (arrows) in the deep white matter of a fetus from an influenza inoculated dam do not autofluorescence under identical conditions (A' and A''). Scale bars: 50 μm.

Figure S4: Gestational Age at Time of Delivery versus Maximal IC Count

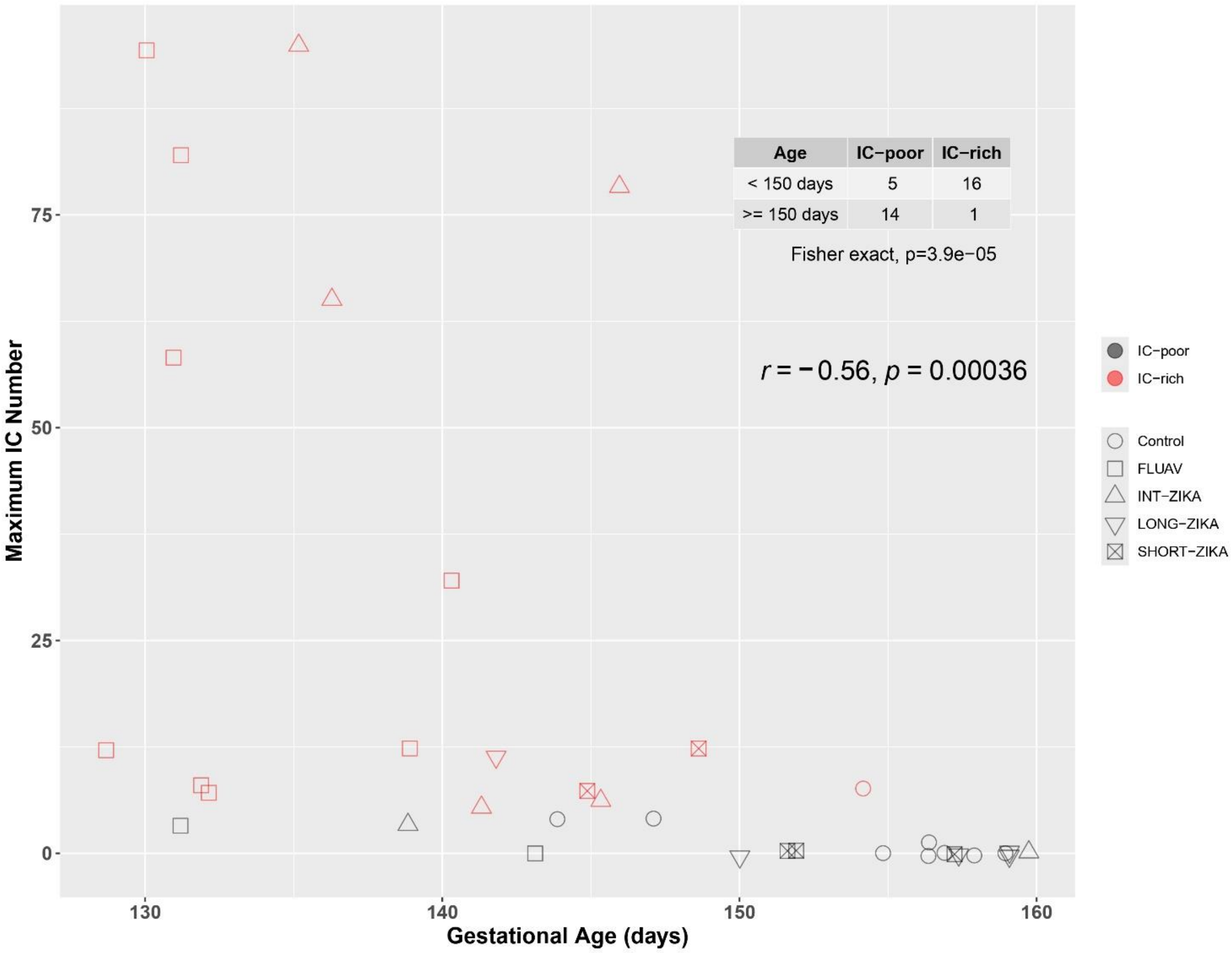

The maximal number of ICs identified in any given coronal section is plotted against the gestational age of the fetus at the time of delivery / necropsy. A strong negative correlation (r=-0.56, p=0.00036) is observed such that ICs were generally more numerous in younger fetuses in this study. All but one (16/17, 94%) of the IC-rich fetuses were delivered at a gestational age of less than 150 days versus 10/19 (53%) of the IC-poor fetuses (Fisher exact, p<0.0001).

Figure S5: Representative Images of LAMP2 Immunohistochemistry

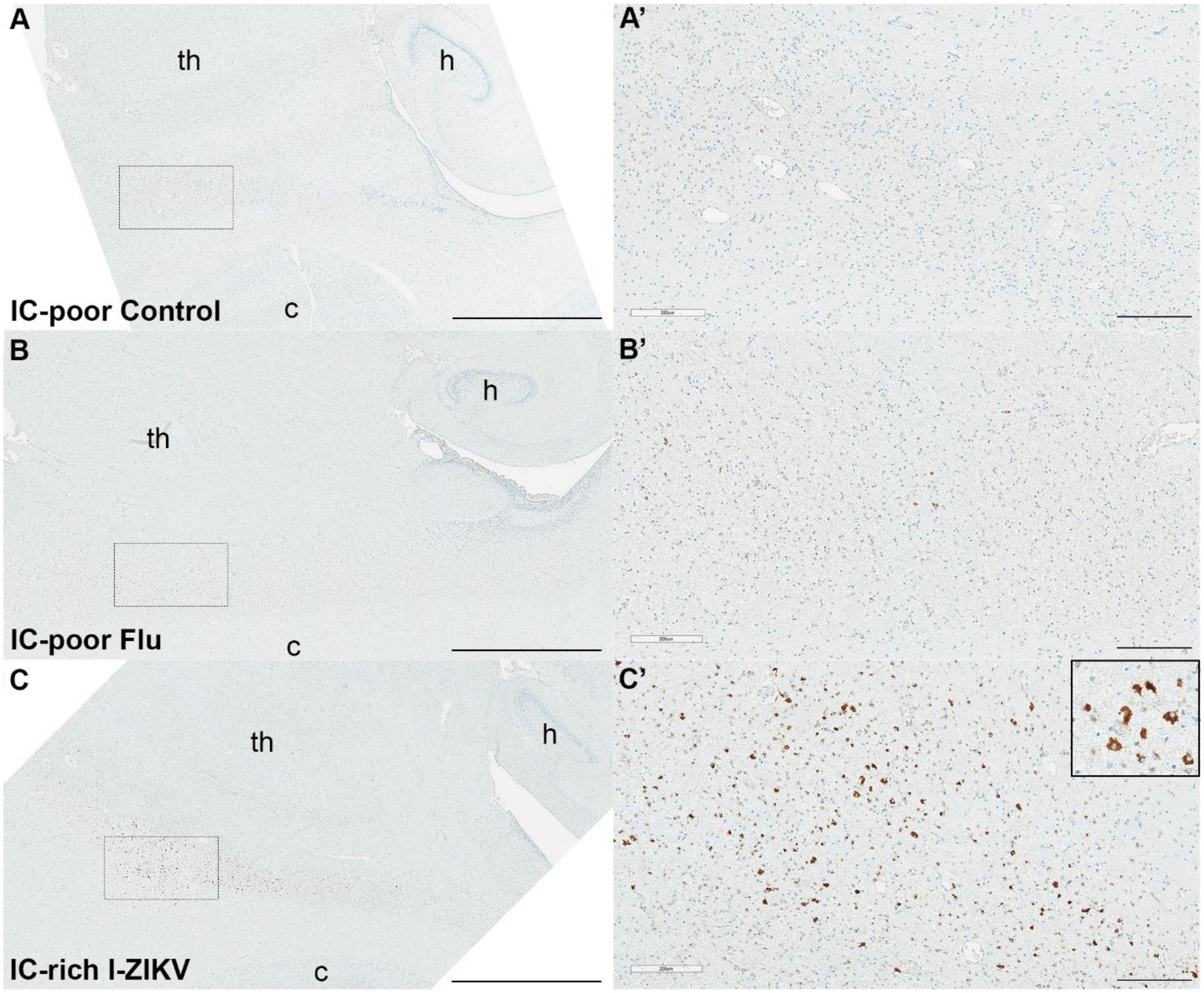

This figure shows LAMP2 immunohistochemistry from IC-poor control (A, A'), IC-poor FLUAV (B, B'), and IC-rich INT-ZIKA (C, C') fetuses. The rectangles in the low magnification images show areas in the ICR, which are shown at higher magnification in A', B' and C'. No and very weak immunolabeling are present in the control and FLUAV ICR's respectively. Intense labeling is present in the ICR of the IC-rich INT-ZIKA brain, including some larger cells (inset) which likely represent inclusion cells. Abbreviations: h, hippocampus; th, thalamus; c, cortex. Scale bars: A-C, 2 mm; A'-C', 200 µm.

Figure S6: Focal Inclusion Cell Aggregates in Thalamus

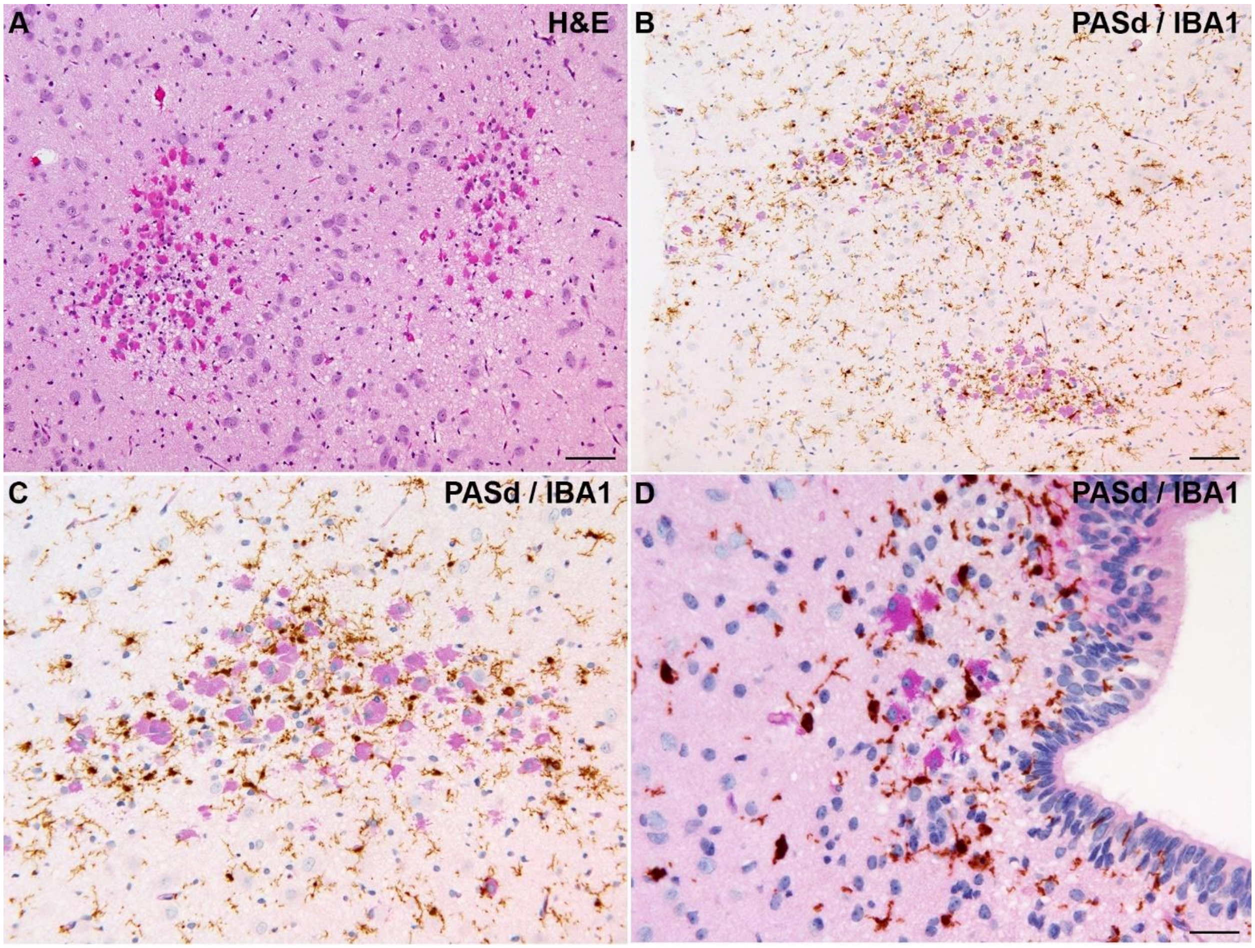

This figure demonstrates aggregates of inclusion cells in the fetal brains of the control (A, B, C) and FLUAV(D) cohorts. (A) Two dense aggregates of inclusion cells in the medial thalamus of one of the control fetuses (H&E, CTRL15). (B,C) The granules in the cytoplasm of these cells are PASd positive and they are surrounded by an increased density of IBA-1-immunoreactive microglial cells. (D) A similar inclusion cell aggregate was present in the medial thalamus of one of the FLUAV fetuses (FLU1). Scale bars: A, 100 µm; B, 100 µm; C, 50 µm; D, 25 µm.

Figure S7: Electron Micrographs of Neuropil with Early Myelination

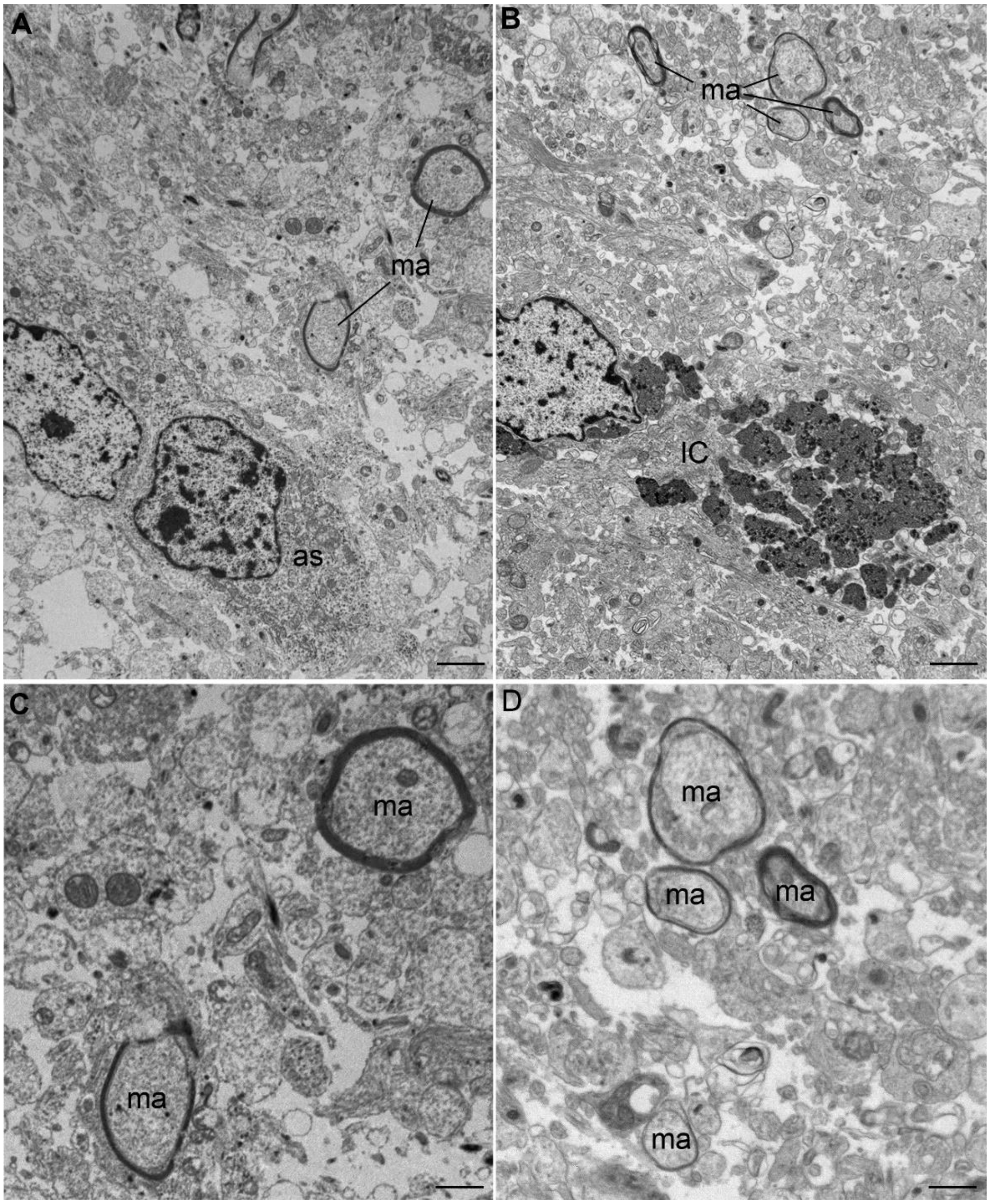

Electron micrographs of deep white matter in inclusion cell region of 147 day of gestation CONTROL 24 (A, C) and 149 day of gestation ZIKA 15 (B, D). Small numbers of myelinated axons are present in the white matter surrounding an astrocyte (as) without inclusions in the control and an inclusion cell (IC) in the fetus from a virus-inoculated dam. Most of the axons in the white matter are not myelinated at this stage of development. Scale bars: A and C, 2 μm; B and D, 1 μm.

Figure S8: Zika Viral Protein NS1 Immunohistochemistry

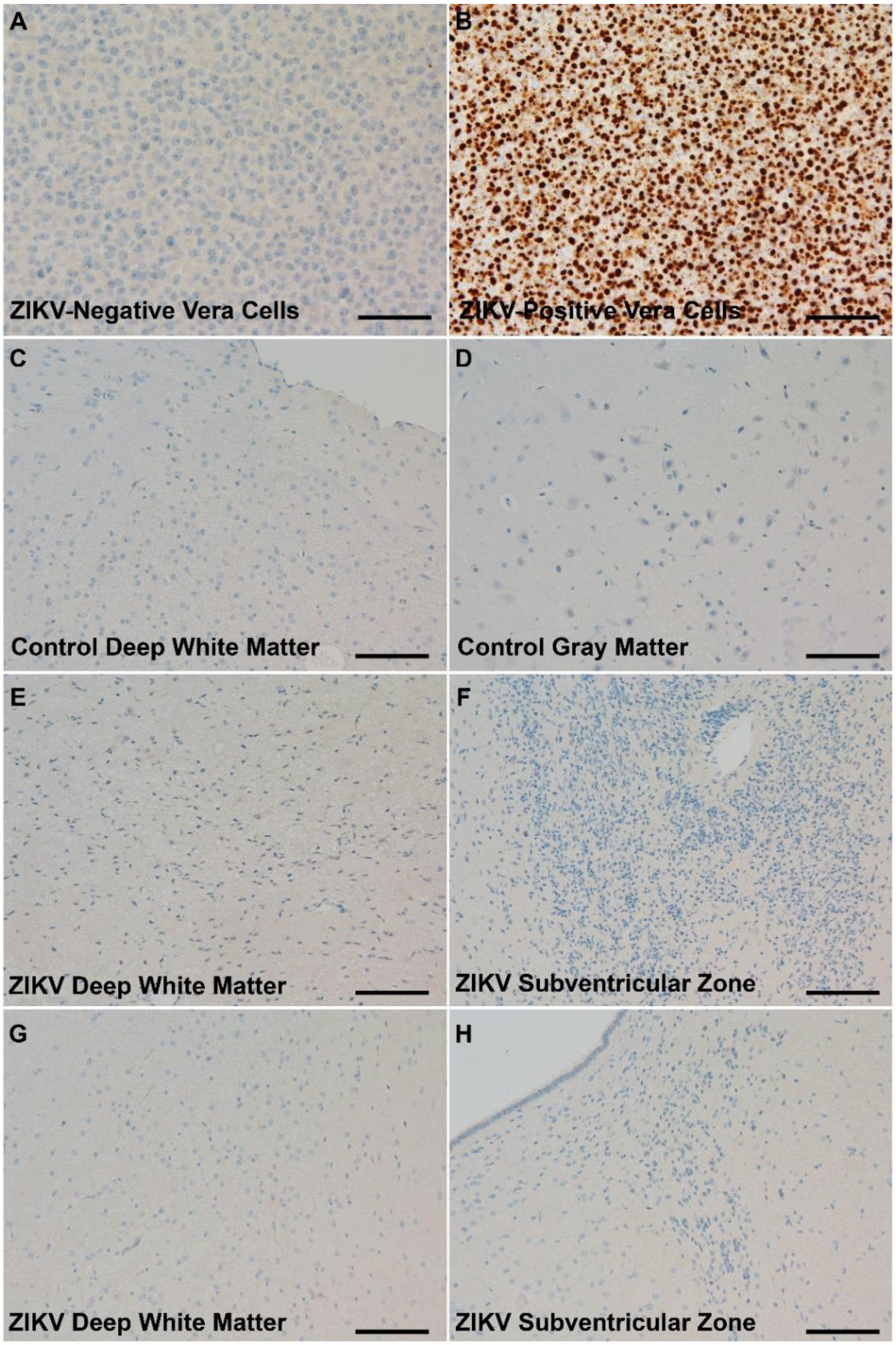

No immunoreactivity is detected in a paraffin section from a pellet of untransfected Vero cells (A), whereas intense diffuse immunolabeling is observed in Vero cells infected with ZIKV (B). (C-F) Brain sections from control (sham inoculation, C, D) and Zika virus-exposed fetuses (E, G) with many inclusion cells show no detectable NS1 immunoreactivity; inclusion cell-rich area is shown in E. Scale bars: A-B, 50 μm; C-H, 100 μm.
